# Facile Synthesis of Atomic Fe‐N‐C Materials and Dual Roles Investigation of Fe‐N_4_ Sites in Fenton‐Like Reactions

**DOI:** 10.1002/advs.202101824

**Published:** 2021-10-12

**Authors:** Jun Wang, Bin Li, Yang Li, Xiaobin Fan, Fengbao Zhang, Guoliang Zhang, Wenchao Peng

**Affiliations:** ^1^ Department of Chemical Engineering Tianjin University Tianjin 300350 China; ^2^ Chemistry and Chemical Engineering Guangdong Laboratory Shantou 515031 China

**Keywords:** atomic Fe‐N‐C materials, degradation mechanism, DFT calculation, dual roles, Fenton‐like reactions

## Abstract

Fenton‐like reactions with persulfates as the oxidants have attracted increasing attentions for the remediation of emerging antibiotic pollutions. However, developing effective activators with outstanding activities and long‐term stabilities remains a great challenge in these reactions. Herein, a novel activator is successfully synthesized with single iron atoms anchored on porous N‐doped carbon (Fe‐N‐PC) by a facile chemical vapor deposition (CVD) method. The single Fe atoms are coordinated with four N atoms according to the XANES, and the Fe‐N_4_‐PC shows enhanced activity for the activation of peroxymonosulfate (PMS) to degrade sulfamethoxazole (SMX). The experiments and density functional theory (DFT) calculations reveal that the introduction of single Fe atoms will regulate the main active sites from graphite N into Fe‐N_4_, thus could enhance the stability and tune the PMS activation pathway from non‐radical into radical dominated process. In addition, the N atoms connected with single Fe atoms in the Fe‐N_4_‐C structure can be used to enhance the adsorption of organic molecules on these materials. Therefore, the Fe‐N_4_‐C here has dual roles for antibiotics adsorption and PMS activation. The CVD synthesized Fe‐N_4_‐C shows enhanced performance in persulfates based Fenton‐like reactions, thus has great potential in the environmental remediation field.

## Graphical abstract

A novel SACs (Fe‐N‐PC) is successfully synthesized by a facile chemical vapor deposition (CVD) method. The introduction of single iron atoms enhances the stability and tune the PMS activation pathway from non‐radical into radical process. In addition, the N atoms connected with iron atoms in Fe‐N_4_‐C structure are proved to be able to enhance the adsorption of organic molecules.

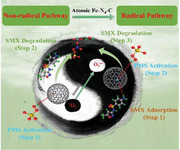

## 1 Introduction

Antibiotics, as a typical member of pharmaceuticals and personal care products (PPCPs), have been defined as a unique group of emerging environmental contaminants. Their treatment is an urgent task due to their abuse and hazardous effects both on human beings and animals even at a low dosage.^[^ ^1^, ^2^ ^]^ Recently, sulfamethoxazole (C_10_H_11_N_3_O_3_S, SMX) has attracted more attention because of its increasing biological resistance and resistant genes.^[^ ^3^ ^]^ Up to now, various strategies have been applied to treat SMX in aquatic environment, such as physical adsorption, membrane filtration, photocatalysis, biodegradation, chlorination, and ozone oxidation.^[^ ^4^, ^5^, ^6^, ^7^, ^8^ ^]^ Regrettably, its effective removal is difficult to achieve via traditional methods owing to its complicated structure, harsh biodegradation conditions, and kinds of toxic degradation intermediates.^[^ ^9^, ^10^ ^]^ Therefore, to develop effective technology to realize the rapid degradation and mineralization of SMX is still a great challenge.^[^ ^1^, ^11^ ^]^

Sulfate radicals (SO_4_^•−^) based advanced Fenton‐like reactions have been regarded as promising candidate for the removal of antibiotics recently.^[^ ^12^ ^]^ During these reactions, various reactive oxygen species (ROSs), such as hydroxyl radicals (^•^OH), sulfate radicals (SO_4_^•−^), superoxide anion (O_2_^•−^), and single oxygen (^1^O_2_) can be generated. Toxic organic contaminants can therefore be degraded into smaller and bio‐degradable molecules, or even into H_2_O and CO_2_.^[^ ^13^, ^14^ ^]^ Compared to other commercial oxidants, such as peroxydisulfates (PDS), H_2_O_2_ and ozone (O_3_), peroxymonosulfates (PMS) is much easier to be activated in a wide pH range due to its asymmetry structure.^[^ ^15^, ^16^ ^]^ According to the previous studies, carbon materials, including carbon nanotubes, graphene, carbon dots, and nano‐diamond could activate PMS through radical or non‐radical process for pollutants degradation.^[^ ^17^, ^18^ ^]^ Furthermore, heteroatoms doping (N, S, B, and P) into these carbon materials can be a feasible approach to increase their activities.^[^ ^13^, ^19^ ^]^ Although with good activities, the poor stability of carbon materials have greatly limited their real application in Fenton‐like reactions.^[^ ^20^ ^]^ PMS can be also activated to achieve degradation of organic pollutants by traditional metal oxides (Fe_2_O_3_, Co_3_O_4_, MnO_2_, MoO_3_, etc.) and spinel metal oxides (CoFe_2_O_4_, CoMn_2_O_4_, etc.).^[^ ^21^, ^22^, ^23^ ^]^ However, large scale application of pure metal compounds will result in secondary pollution easily or produce large amounts of sludge.^[^ ^24^ ^]^

Single‐atom materials are emerging as potential alternatives in multiple catalytic reactions, owing to utmost atomic utilization and outstanding catalytic efficiency.^[^ ^25^, ^26^, ^27^, ^28^ ^]^ For the synthesis of single‐atom materials, the most critical point is to provide an excellent coordination environment to prevent metal aggregation.^[^ ^29^, ^30^ ^]^ Single metal atoms coordinated by nitrogen and carbon (M‐N‐C, M = Pt, Pb, Fe, Co, Mn, Cu, etc.) have been reported to be a widely used strategy to avoid clusters or larger particles forming.^[^ ^31^, ^32^ ^]^ Considering the coexistence of single metal atoms and nitrogen doped carbons, the single atoms M‐N‐C materials should have both high catalytic activity and excellent stability in Fenton‐like reactions.^[^ ^33^, ^34^, ^35^, ^36^ ^]^ However, an accurate judgment of reactive sites and mechanism for PMS activation on single atoms M‐N‐C materials are still absent and need to be deeply and systematically investigated.

In this study, single‐atom Fe‐N_4_‐PC materials are fabricated by a chemical vapor deposition (CVD) method, and both characterization and theoretical calculation suggest that each single Fe atom is anchored by four N atoms. The obtained materials are applied to activate PMS for the degradation of SMX, and the dual roles of Fe‐N_4_ sites are also investigated. Free radical quenching experiments and EPR tests are used to reveal the roles of generated reactive oxidative species (ROSs) in this system. This study aims to show the details of active sites and PMS activation mechanism on the single‐atom Fe‐N‐C materials, thus could promote the real application of them in environmental remediation processes.

## 2 Results and Discussion

### 2.1 Synthesis and Structural Characterization

**Figure 1:**
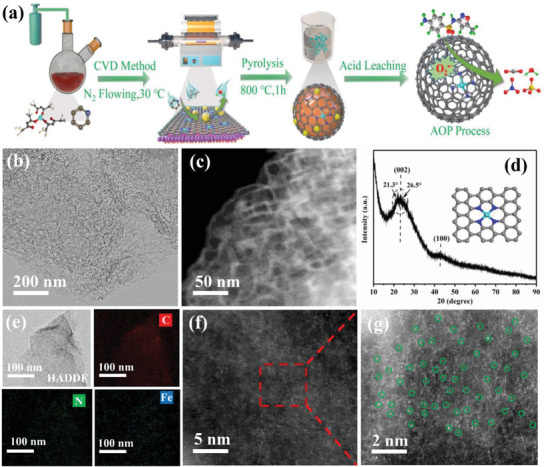
The synthesis process and morphology characterization. Schematic for the synthesis of a) Fe‐N_4_‐PC‐2 materials, b) HRTEM image, c) HAADF‐STEM image, d) XRD pattern, e) EDS mapping images, f) Aberration‐corrected HAADF‐STEM image, and g) enlarged image of single Fe atom in green circles.

The synthesis of single‐atom Fe‐N‐C materials using a chemical vapor deposition (CVD) method is illustrated in **Figure** **1**a. Iron (III) acetylacetonate dissolved in pyridine is used as precursors and is bubbled into the CVD system filled with Mg(OH)_2_ template. Afterward, the Mg(OH)_2_ template and iron oxide with large diameters were etched by 1 m H_2_SO_4_ solution. The obtained samples are denoted as Fe‐N_4_‐PC‐1, Fe‐N_4_‐PC‐2, Fe‐N_4_‐PC‐3 with the pyrolysis temperatures at 700, 800, and 900°C, respectively.

High resolution transmission electron microscopy (HR‐TEM) reveals that Fe‐N_4_‐PC‐2 is in a thin and lamellar graphitized carbon structure with abundant mesopores (Figure 1b,c). Nitrogen adsorption result of Fe‐N_4_‐PC‐2 shows a typical type IV isotherm with a hysteresis loop, further demonstrating its mesoporous structure (Figure S1 and Table S1, Supporting Information). The X‐ray diffraction (XRD) spectroscopy of Fe‐N‐C‐2 is shown in Figure 1d, and only two broad diffraction peaks (002 and 100) are found, corresponding to the characteristic planes of amorphous carbon (Figure S2a,b: Supporting Information). Notably, the bump between 21.3° and 26.5° suggests that the obtained material is long‐range disordered. There is no other obvious metallic peak could be detected in Fe‐N_4_‐PC‐2, indicating that crystalline Fe containing species are not formed or have been removed after acid leaching. In addition, the Raman spectra has been used to investigate the defect degree. As displayed in Figure S3 (Supporting Information), there is a defect site‐related D band at 1335 cm^−1^ and a sp^2^ hybridized carbon correlated G band at 1587 cm^−1^ for Fe‐N_4_‐PC‐X samples. With thermal annealing temperature increasing from 600 to 800°C, the calculated *I*_D_/*I*_G_ (intensity ratios of D band to G band) ranges from 1.21 to 1.42 for the Fe‐N_4_‐PC‐X samples. The increased *I*_D_/*I*_G_ indicates the enrichment of defective structure and declined sp^2^ carbon. To further determine the Fe content of Fe‐N_4_‐PC‐2, inductively coupled plasma atomic emission spectrometry (ICP‐AES) is investigated, and the weight percentage of Fe anchored on the carbon material is 1.23 wt%. Then, the atomically dispersed Fe species is elucidated by high‐angle annular dark‐field scanning transmission electron microscopy (AC HAADF‐STEM) measurements according to the bright dots of atomic size. As shown in HAADF‐STEM and corresponding EDX mapping images (Figure 1e), the C, N, and Fe elements are distributed uniformly in Fe‐N_4_‐PC‐2. The highly dispersed sparking points without obvious aggregation confirm the single‐atom feature of Fe (Figure 1f,g and Figure S4a–d: Supporting Information). All these results are in accordance with the previously mentioned XRD, EDX mapping and AC HAADF‐STEM images, indicating that Fe is atomically dispersed in the carbon layers.

**Figure 2:**
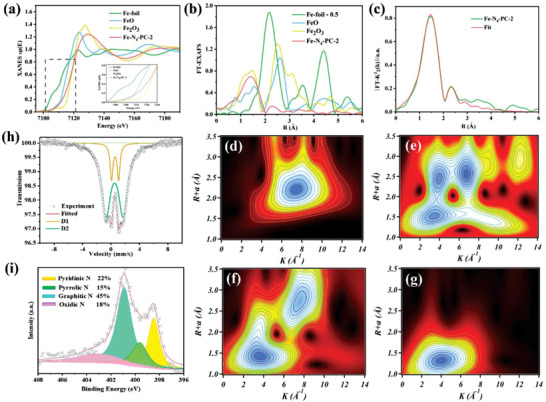
Chemical environment of Fe‐N_4_‐PC‐2 catalyst. a) Fe K‐edge XANES spectra of Fe foil, FeO, Fe_2_O_3_, and Fe‐N_4_‐PC‐2 (inset was the magnified image), b) The Fourier transformed (FT) k^3^‐weighted EXAFS, c) EXAFS fitting curves of Fe‐N_4_‐PC‐2 at R space, d–g) WT of the Fe K‐edge (d‐Fe, e‐FeO, f‐Fe_2_O_3_, g‐ Fe‐N_4_‐PC‐2), h) The ^57^Fe Mössbauer spectrum measured at 25 °C, i) N 1s XPS spectra.

X‐ray absorption fine structure (XAFS) and X‐ray absorption near‐edge structure spectra (XANES) are conducted to demonstrate the existence of possible atomic structures of the samples. As shown in **Figure** **2**a, the position of Fe K‐edge XANES spectra for Fe‐N_4_‐PC‐2 is located between FeO and Fe_2_O_3_, indicating that the valence of Fe in Fe‐N_4_‐PC‐2 is in the range of +2–+3. The formation of Fe−N bonds in Fe‐N_4_‐PC‐2 is confirmed by the Fourier transform (FT) k^3^‐weighted EXAFS in Figure 2b. The obvious peak at 1.4 Å of Fe‐N_4_‐PC‐2 corresponds to the Fe—N bond, which extremely distinguishes Fe–Fe coordination peak at 2.2 Å in Fe foil, FeO and Fe_2_O_3_ samples, manifesting the atomic Fe disperses on Fe‐N_4_‐PC‐2. According to the least‐squares EXAFS fitting curves and fitting results, the first shell of the Fe atom in Fe‐N_4_‐PC‐2 is coordinated with 4 N atoms (Figure 2c and Figures S5 and S6a–f and Table S2: Supporting Information). The Wavelet Transform (WT) contour plots of Fe foil exhibit one intensity maximum of Fe–Fe signal at about 7.8 Å^−1^, while those of Fe‐N_4_‐PC‐2 only exhibit one intensity maximum at about 4 Å^−1^, which is associated with the Fe—N bond, further proving the atomically dispersion of Fe atoms (Figure 2d–g). The presence of Fe–N_x_ moieties is also supported by ^57^Fe Mössbauer spectra collected at room temperature (Figure 2h and Table S3: Supporting Information). The spectrum of the Fe‐N_4_‐PC‐2 sample can be fitted into two doublets D1 and D2, whose area proportions are calculated to be 84% and 16%, respectively. The doublet D1 has been widely observed for Fe–N–C materials and recently is identified as distorted Fe^II^–N_4_‐C intermediate spin, while D2 is assigned to be an Fe^II^–N_4_ high spin site. The oxidation and spin state for both of them will keep unchanged under different electrochemical potentials. Combining the relevant results of XAFS, XANES and Mössbauer tests, the state of Fe in Fe‐N_4_‐PC‐2 is in the form of Fe‐N_4_ structure. XPS survey spectra and the N 1s spectra of these samples reveal the coexistence of four types N species, including pyridinic N (398.4 eV), pyrrolic N (399.6 eV), graphitic N (400.8 eV), and oxidized N (403.1 eV) (Figure 2i, Figures S7, S8a–d and Table S4: Supporting Information). The abundant N in the carbon network is facile for Fe to be anchored as single atom. Figure S9 (Supporting Information) summarized all the 5 binding states of N in Fe‐N_4_‐PC‐2 single‐atom catalyst.

### 2.2 Persulfate Activation Performance

**Figure 3:**
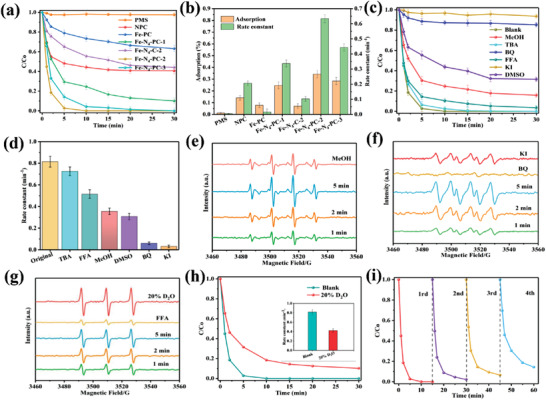
Catalytic degradation test and mechanism analysis. a) The removal of SMX in different reaction systems, b) The relationship between adsorption ability and the reaction rate constants on different catalysts, c) Inhibition effect of different quenchers on SMX degradation in Fe‐N_4_‐PC‐2/PMS system, d) Comparison of reaction rate under different quenching conditions, e) EPR spectrum of DMPO‐^•^OH and DMPO‐SO_4_^•−^ radicals, f) EPR spectrum of DMPO‐O_2_^•−^ radical, g) EPR spectrum of TMPO‐^1^O_2_ radical, h) The effect of reaction solvents (H_2_O and D_2_O) upon degradation (inset was the reaction rate constants), i) Catalyst recyclability tests. Reaction condition: SMX concentration = 10 ppm, PMS concentration = 0.30 × 10^−3^ m, Catalyst dosage = 30 mg L^−1^, Reaction temperature = 298 K, and initial pH value = 6.0.

The obtained single atom Fe‐N‐C samples are then evaluated to activate PMS for SMX degradation. As shown in **Figure** **3**a, without the addition of any activator, PMS can hardly degrade SMX in 30 min. Without the introducing of single Fe atoms, NPC and Fe‐PC can only obtain SMX removal efficiency of 59.1% and 36.7%, respectively. Surprisingly, 100% SMX could be degraded in 15 min with Fe‐N_4_‐PC‐2 as activator, indicating the key role of the single Fe atoms for PMS activation. It should be noted that the activity of Fe‐N_4_‐PC‐2 for SMX degradation has been by far the most active among all the reported materials (Table S4, Supporting Information). In addition, both annealing temperature and template have great influence on the performance of the synthesized activators. Fe‐N_4_‐PC‐1, Fe‐N_4_‐C‐2, and Fe‐N_4_ ‐PC‐3 could achieve 89.6%, 56.3%, and 99.7% SMX removal within 30 min, respectively. N_2_ adsorption‐desorption isotherms confirm that Mg(OH)_2_ template has a larger specific surface area and more uniform pore size distribution than MgO (Figure S10a,b: Supporting Information). Furthermore, the generation of H_2_O produced from Mg(OH)_2_ decomposition could create porous structure, resulting in the enhanced activity of Fe‐N_4_‐PC‐2. Interestingly, the order of the reaction rate constants is found to match well with the adsorption abilities for these materials (Figure 3b). Particularly, Fe‐N_4_‐PC‐2 presents the best capacity for organics adsorption among these materials, and almost 34.3% of SMX can be absorbed when reaching the dynamic adsorption‐equilibrium stage, indicating the outstanding adsorption capacity of Fe‐N_4_‐PC‐2 for SMX.

The performances of Fe‐N_4_‐PC‐2 under different test conditions are further carried out. It could be seen form Figure S11a (Supporting Information) that the SMX degradation efficiencies increase with the Fe‐N_4_‐PC‐2 dosage. As for the effect of PMS dosage, SMX degradation efficiency is enhanced with the PMS addition amount increasing from 0.1 to 0.4 × 10^−3^ m, but an efficiency decrease could be observed with a PMS loading of 0.5 × 10^−3^ m, probably due to the self‐quenching effect (Figure S11b, Supporting Information). The activation performances with different SMX concentrations from 2 to 15 ppm are illustrated in Figure S11c (Supporting Information). Even with the concentration of 15 ppm, 100% degradation of SMX can be accomplished within 30 min, indicating the outstanding activation ability of Fe‐N_4_‐PC‐2. The influence of initial reaction pH on SMX degradation is also detected for the Fe‐N_4_‐PC‐2/PMS system (Figure S11d, Supporting Information). With the initial pH decreasing from 8 to 2, Fe‐N_4_‐PC‐2 could keep its high activity very well, but a higher pH >10 will cause the remarkable deactivation. The influence of solution pH can be well explained by zero point of charge (pH_ZPC_). According to the results in Figure S12a‐b (Supporting Information), the pH_ZPC_ points of NPC and Fe‐N_4_‐PC‐2 are around 7.144 and 7.318, respectively. A higher pH (>10) would hinder the PMS adsorption and activation, thus slowing down the reaction rates.

Usually, some anions, such as Cl^−^, H_2_PO_4_^−^, NO_3_^−^, and HCO_3_^−^ are extensively existed in actual industrial wastewater matrix, which can interact with some free radicals, especially hydroxyl radical and sulfate radical, resulting in the reduction of degradation efficiency. (Text S1, Supporting Information). Hence, the effects of these anions are then investigated in detail for further confirmation. Surprisingly, the existence of these anions in water has a slight effect on SMX degradation even at high concentrations, indicating that PMS activation in the Fe‐N_4_‐PC‐2/PMS system is not dominated by hydroxyl radical or sulfate radicals (Figure S13a–d: Supporting Information). The degradation performances of Fe‐N_4_‐PC‐2 for other organic pollutants are also studied. As shown in Figure S14 (Supporting Information), Fe‐N_4_‐PC‐2 could also activate PMS to completely degrade other common organic pollutants, such as Rhodamine B (RhB), Bisphenol A (BPA), 2,4‐Dichlorophenol (2,4‐DCP), and phenol. Even for the refractory Tetracycline (TC), 89.7% removal could be achieved within 30 min, indicating the excellent activity and adaptability of Fe‐N_4_‐PC‐2 for various contaminants. The degradation performances of SMX with different oxidants are also tested for a comparison (Figure S15, Supporting Information). With PDS, O_3_, and H_2_O_2_ as the oxidants, 88.9%, 65.1%, and 19.8% of SMX removal could be achieved within 30 min, respectively. These results demonstrate that PMS is the most suitable oxidant in the Fe‐N_4_‐PC‐2/SMX system.

### 2.3 Activation Mechanism

In order to identify the reactive oxidative species (ROSs) generated during PMS activation in the Fe‐N_4_‐PC‐2/PMS system, radical quenching tests are then conducted. Typically, methanol (MeOH) is used as the scavenger for both hydroxyl radical (^•^OH) and sulfate radical (SO_4_^•−^), while tert‐butanol (TBA) is just used as the scavenger for hydroxyl radical (^•^OH). The reaction rate constants of different scavengers with the target ROSs are summarized in Table S5 (Supporting Information).^[^ ^36^, ^37^, ^38^, ^39^ ^]^ As shown in Figure 3c, with TBA added into the reaction mixture, the SMX degradation rate is decreased slightly, indicating the weak contribution of •OH. MeOH has better inhibition effect than TBA, proving the presence of SO_4_^•−^. Furfuryl alcohol (FFA) is usually used to selectively screen ^1^O_2_ radical, which also shows better inhibition than TBA. When benzoquinone is added to quench the superoxide anion radical (O_2_^•−^), SMX removal efficiency drops drastically, thus proving the crucial role of O_2_^•−^ in the Fe‐N_4_‐PC‐2/PMS system. To verify the dominant role of O_2_^•−^ radical, the contribution of •OH, SO_4_^•−^, O_2_^•−^, and ^1^O_2_ are compared quantitatively according to the reported method.^[^ ^14^ ^]^ As shown in Figure 3d, the rate constants with the addition of MeOH, TBA and FFA, BQ are determined to be 0.815, 0.3554, 0.7257, 0.0596, and 0.5153 min^−1^, respectively. Therefore, the individual contribution of •OH, SO_4_^•−^, O_2_^•−^, and ^1^O_2_ are calculated to be 3.1%, 12.1%, 76.2%, and 8.6% respectively, indicating the dominant role of O_2_^•−^ radical in the Fe‐N_4_‐PC‐2/PMS system. In addition, DMSO and KI are used to quench the surface‐bounded ROSs, and the degradation efficiencies of SMX are declined from 100% to 68.5% and 6.4% in 30 min after the addition of DMSO and KI, respectively. The added KI can be oxidized into I_2_, which can shield the interaction between organic molecules and the catalyst surface, thereby hindering the occurrence of surface reactions. The strong inhibition of DMSO and KI suggest that the surface activated ROSs play a remarkable role when using Fe‐N_4_‐PC‐2 as catalyst for SMX degradation.

In addition, electron paramagnetic resonance (EPR) is also used to further verify the ROSs in the Fe‐N_4_‐PC‐2/PMS system with 5,5‐Dimethyl‐1‐pyrroline N‐oxide (DMPO) and 2,2,6,6‐tetramethyl‐4‐piperidinol (TEMP) as spin trapping agents. DMPO can be used as spin trapping agents for •OH, SO_4_^•−^ and O_2_^•−^ radicals, while TEMP can be used as specific spin trapping agent for ^1^O_2_. As depicted in Figure 3e, when DMPO is injected into the reaction system, the existence of DMPO‐^•^OH and DMPO‐SO_4_^•−^ can be verified in this system. As shown in Figure 3f, six‐line characteristic peaks of DMPO‐O_2_^•−^ are observed. This result could prove that O_2_^•−^ is produced with the addition of Fe‐N_4_‐PC‐2, which agrees well with the quenching tests. Furthermore, the peak intensity of O_2_^•−^ had a dramatically decline with the addition of BQ and KI. Besides, TEMP can be used as specific spin trapping agent for ^1^O_2_. The Characteristic signals are captured in the existence of TEMP (Figure 3g), evidencing the participation of ^1^O_2_ in the Fe‐N_4_‐PC‐2/PMS system. Similarly, the signal of ^1^O_2_ will disappear with FFA as the quencher. Although ^•^OH, SO_4_^•−^ and ^1^O_2_ radicals didn't have direct inhibitory ability, the existence and accumulation of these radicals will promote the formation of O_2_^•−^ through mutual transformation of different radicals, resulting in the acceleration of SMX degradation (Text S2, Supporting Information).

Typically, ^1^O_2_ could be generated from the self‐decomposition of PMS and the mutual conversion with O_2_^•−^ (Text S6, Supporting Information).^[^ ^40^, ^41^ ^]^ In order to prove the conclusion of the quenching experiments further, we also used D_2_O as the solvent to perform the quenching experiments. The lifetime of ^1^O_2_ is closely related to the solvent, and the relevant data are listed in Table S6 (Supporting Information). It can be seen that the lifetime of ^1^O_2_ in D_2_O (20−32 µs) is 10 times longer than that in H_2_O (2 µs). The accumulation of ^1^O_2_ can therefore be accelerated in D_2_O solvent, while less O_2_^•−^ will be left. This can be confirmed by the EPR results in Figure 3g, where the signal of ^1^O_2_ is enhanced obviously after the using of D_2_O as solvent. According to the quenching results in Figure 3c,d,f, O_2_^•−^ is more effective than ^1^O_2_ for SMX degradation in the Fe‐N_4_‐PC‐2/PMS system. As shown in Figure 3h, the SMX degradation is obviously decelerated with D_2_O addition. It can be therefore proved that some O_2_^•−^ with more effective degradation ability has been transformed into ^1^O_2_ in this system, thus resulting in the slowly degradation rate. The using of D_2_O as solvent could prove the above quenching experiment results further.

**Figure 4:**
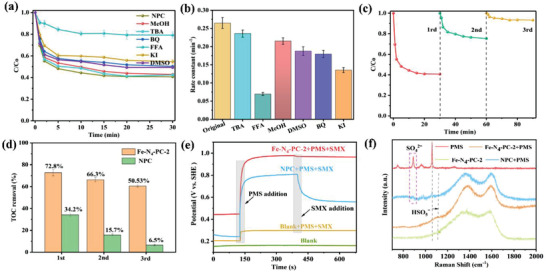
The PMS activation route transition from non‐radical to free radical process with single Fe atoms introduced in the Fe‐N_4_‐PC‐2/PMS system. a) The influence of different quenchers on SMX degradation in NPC/PMS system, b) Comparison of reaction rate under different quenching conditions, c) Catalyst recyclability tests in NPC/PMS system, d) TOC removal, e) Open‐circuit potential curves, and f) In situ Raman spectra. Reaction condition: SMX concentration = 10 ppm, PMS concentration = 0.30 × 10^−3^ m, Catalyst dosage = 30 mg L^−1^, Reaction temperature = 298 K, and initial pH value = 6.0.

The introduction of single atoms will greatly improve the stability of the carbon catalyst, which could also tune the PMS activation route. As shown in **Figure** **4**a‐b, pure N‐doped carbon material (NPC) shows weaker PMS activation ability than sing‐atom catalyst (Fe‐N_4_‐PC‐2). Less than 60% of SMX could be degraded within 30 min in NPC/PMS system. Furthermore, quenching experiments indicated that ^1^O_2_ is the main active species in the NPC/PMS system, rather than O_2_^•−^ radical. The carbon‐based catalyst (NPC) exhibits extremely poor stability in SMX degradation (Figure 4c). Interestingly, stability of the catalyst is greatly improved after the introduction of single Fe atoms (Figure 3i). Even after four cycles, the catalytic ability of the catalyst only decreases slightly. According to our previous work, the deactivation of the material is attributed to electron transfer and carbon oxidation. The related schematic diagram is shown in Figure S16 (Supporting Information). ICP‐MS and XPS results show that the content of Fe in Fe‐N_4_‐PC‐2 is basically unchanged after stability test (Table S7, Supporting Information). However, related oxygen‐containing groups is greatly increased. In order to remove these oxygen‐containing groups, a high temperature annealing process is carried out, and the activity of Fe‐N_4_‐PC‐2 can be significantly recovered after annealing treatment (Figure S17, Supporting Information). The removal of oxygen‐containing groups on adjacent carbon atoms promotes the occurrence of electron transfer processes, which in turn promotes the activation of PMS. Compared with other reported N‐doped carbon and Fe‐based materials, Fe‐N_4_‐PC‐2 in this work also has satisfying performance (Table S8, Supporting Information). Therefore, the introduced single‐atom Fe could make full use of the electrons on the adjacent carbons, thus achieving a much better stability than the metal free carbons.

Moreover, electrochemical techniques can be used to investigate the contribution of non‐radical process. Among various testing methods, the open‐circuit potential (OCP) test is widely used in Fenton‐like reactions because it can more accurately and directly reveal the electron transfer.^[^ ^20^, ^42^, ^43^ ^]^ As shown in Figure 4d, OCP of NPC‐GCE electrode is increased immediately after PMS addition. It is speculated that the formation of NPC/PMS complex could elevate the potential of NPC. The obvious potential decrease with SMX added could indicate the electron‐transfer process (non‐radical process) from SMX to the NPC/PMS complex.^[^ ^44^ ^]^ Hence, non‐radical process plays a very important role with NPC as the catalyst. In contrast, the potentials of Fe‐N_4_‐PC‐2‐GCE electrode with or without SMX addition show no obvious difference, implying the weak non‐radical process in the Fe‐N_4_‐PC‐2/PMS system. This result could further prove the PMS activation routes is transformed from non‐radical into radical process after the introduction of single‐atom Fe. In addition, in situ Raman technology was conducted (Figure 4e). The blue‐shift of HSO_5_^−^ characteristic vibration peak from 1062 to 1116 cm^−1^ indicates the stretch of O—O bond, resulting in the activation of PMS.

For the Fe‐based Fenton‐like reactions, the important role of high‐valent Fe species (Fe (IV = O)) has been reported.^[^ ^45^, ^46^, ^47^ ^]^ As the probe reaction, methyl phenyl sulfoxide (PMSO) can be oxidized by high‐valent Fe species to form dimethyl sulfone (PMSO_2_) via an oxygen‐atom‐transfer process.^[^ ^48^, ^49^ ^]^ The transformation from PMSO to PMSO_2_ can be monitored by HPLC, the existence of high‐valence Fe can thus be confirmed. As shown in Figure S18 (Supporting Information), PMSO cannot be oxidized by PMS alone or with NPC as catalyst. With the addition of Fe‐N_4_‐PC‐2, 80% of PMSO (20 × 10^−6^ m) can be transformed in 30 min. However, the HPLC peak of generated PMSO_2_ will disappear with further oxidation, indicating that PMSO may be broken down into smaller molecules by certain active species. Usually, in a system dominated by high‐valent Fe, the oxidation process will be ended with PMSO converted into PMSO_2_, and further degradation will not happen.^[^ ^50^, ^51^, ^52^ ^]^ Therefore, we speculate that this process is not dominated by high‐valence Fe.

Ideally, SMX could be degraded and finally mineralized into CO_2_ and H_2_O in the Fe‐N_4_‐PC‐2/PMS system. Therefore, Total Organic Carbon (TOC) measurement is conducted to investigate the mineralization degree, which is an important basis for evaluating the organic contents in the wastewater.^[^ ^53^ ^]^ As described in Figure 4f, >72.8% and 34.2% of TOC removal could be achieved in the Fe‐N_4_‐PC‐2/PMS and NPC/PMS system in the first cycle respectively, indicating the better mineralization ability of single‐atom Fe‐N_4_‐PC‐2 than NPC. In order to further explore the detailed degradation process of SMX in Fe‐N_4_‐PC‐2/PMS system, intermediate products are further determined by High Performance Liquid Chromatography (LC‐MS). As summarized in Table S9 and Figures S19–S20 (Supporting Information), the oxidation of SMX could be divided into three stages. Firstly, the SMX is oxidized to form single‐ring organic compounds, which would be then degraded into ring‐opened molecules. Finally, most of these organic molecules can be further mineralized into NO_3_^−^, CO_2_, or H_2_O. Based on the quenching radicals process, EPR tests, and LC‐MS analysis, the related mechanism of free radical generation and function is shown in Equations (1–10).

(1) $$\begin{array}{ccc} & & \mathrm{Fe}\left(\mathrm{low}\;\mathrm{valence}\right)-N_{4}+{\mathrm{HSO}}_{2}^{-}\to \mathrm{Fe}\left(\mathrm{high}\;\mathrm{valence}\right)\\ & & \;-N_{4}+{\mathrm{SO}}_{4}^{\cdot -}+OH^{-} \end{array}$$

(2) $$\begin{array}{ccc} & & \mathrm{Fe}\left(\mathrm{high}\;\mathrm{valence}\right)-N_{4}+{\mathrm{HSO}}_{5}^{-}\to \mathrm{Fe}\left(\mathrm{low}\;\mathrm{valence}\right)\\ & & \;-N_{4}+{\mathrm{SO}}_{5}^{\cdot -}+H^{+} \end{array}$$

(3) $${\mathrm{SO}}_{4}^{\cdot -}+H_{2}O\to {\mathrm{SO}}_{4}^{2-}+\cdot \mathrm{OH}+H^{+}$$

(4) $${\mathrm{HSO}}_{5}^{-}\to H^{+}+{\mathrm{SO}}_{5}^{2-}$$

(5) $${\mathrm{SO}}_{5}^{2-}+H_{2}O\to H_{2}O_{2}+{\mathrm{SO}}_{4}^{2-}$$

(6) $$\cdot \mathrm{OH}+H_{2}O_{2}\to {\mathrm{HO}}_{2}^{\cdot }+H_{2}O$$

(7) $${\mathrm{HO}}_{2}^{\cdot }\to O_{2}^{\cdot -}+H^{+}$$

(8) $${2O}_{2}^{\cdot -}+2H_{2}O\to {}^{1}O_{2}+H_{2}O_{2}+2OH^{-}$$

(9) $$\cdot \mathrm{OH}+O_{2}^{\cdot -}\to {}^{1}O_{2}+OH^{-}$$

(10) $$\mathrm{SMX}+O_{2}^{\cdot -}\to \mathrm{intermediates}\to CO_{2}+H_{2}O$$

### 2.4 Theoretical Calculation

**Figure 5:**
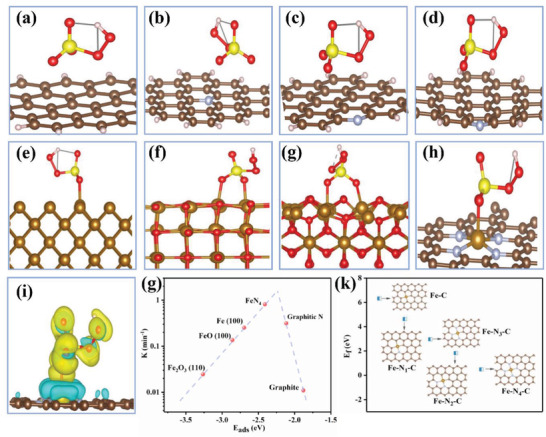
The activation mechanism of PMS on single‐atom Fe‐N_4_‐PC‐2 catalyst. Optimized configurations of PMS adsorbed on a) Graphite, b) Graphitic N, c) Pyridinic N, d) Pyrrolic N, e) Fe (100), f) FeO (100), g) Fe_2_O_3_ (110), and h) Fe‐N_4_ ‐graphene structure, respectively. i) Charge density distribution of Fe‐N_4_‐PC‐2/PMS system. g) Volcano curve. k) The formation energies of different single‐atom Fe coordination models.

DFT calculations are then used to investigate the activation mechanism over Fe‐N_4_‐PC‐2/PMS system (Text S3, Supporting Information). **Figure** **5**a–h (side view) and Figure S21a−h (Supporting Information) (top view) show the optimized configurations of PMS adsorbed on graphite, graphitic N, pyridinic N, pyrrolic N, Fe (100), FeO (100), Fe_2_O_3_ (110), and single‐atom Fe‐N_4_‐C structure, respectively. According to the calculated adsorption energies (E_ads_) summarized in Table S10 (Supporting Information), the adsorption of PMS on the surface of a single atom has competitive adsorption energies (E_ads_) and electron transfer (Q) ability, resulting in a longer O—O bond and PMS activation. Additionally, as evidenced from the charge density analysis (Figure 5i), there exists significant electron transfer between PMS and FeN_4_, reflecting the chemisorption of PMS on the FeN_4_ site. Interestingly, it could be seen from Figure 5g that the adsorption energy of PMS and related the reaction rate follow a volcano relation. Compared with various carbon materials, metallic iron and iron oxide, Fe‐N_4_‐PC‐2 shows the best catalytic ability for PMS activation and SMX degradation. Figure 5k illustrates the formation energies of different single‐atom Fe coordination models (Fe‐C, Fe‐N_1_‐C, Fe‐N_2_‐C, Fe‐N_3_‐C, and Fe‐N_4_‐C). The formation possibility and stability of different structure is closely related to its formation energy. It could be found that central Fe surrounded by four N atoms possess the lowest formation energy, indicating the stable property of the Fe‐N_4_‐C structure.

**Figure 6:**
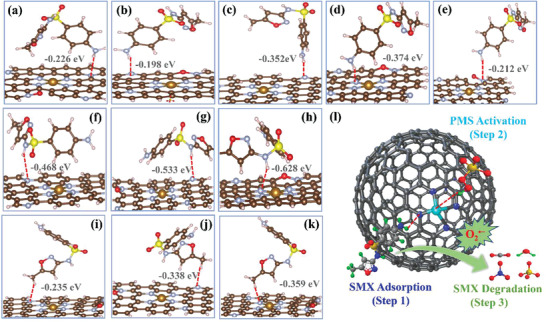
SMX adsorption and degradation process on single‐atom Fe‐N_4_‐PC‐2 catalyst. Adsorption simulation of amino (‐NH_2_) on a) Graphitic N, b) Pyridinic N, c) Pyrrolic N, d) Fe‐N_4_ ‐graphene, and e) Oxide N structure. Local adsorption configurations of imino (‐NH‐) on f) Graphitic N, g) Pyrrolic N and h) Fe‐N_4_‐C structure. Local adsorption configurations of methyl (‐CH_3_) on i) Graphitic N, j) Pyrrolic N and k) Fe‐N_4_ ‐C structure. l) The proposed overall degradation mechanism in the Fe‐N_4_‐PC‐2/PMS system.

According to the adsorption test, it could be found that the introduction of single atom can greatly enhance the adsorption capacity toward SMX. In previous studies, pyrrolic N was proposed to be the adsorption sites, which was associated with the adsorption capacities of those N‐doped carbons toward the organic contaminants^[^ ^30^, ^54^ ^]^ In this study, there is however no clear relationship between pyrrolic N content and SMX adsorption capacity in the Fe‐N_4_‐PC‐2/PMS system. Therefore, the adsorption of SMX by various functional groups onto different types of N are calculated. Firstly, the van der Waals forces between amino (‐NH_2_) and various types of N atoms are analyzed (**Figure** **6**a–e and Table S11: Supporting Information). Interestingly, the adsorption of SMX on Fe‐N_4_‐C structure is proved to be with the shortest H‐N distance (1.999 Å) and the most stable adsorption energy (−0.374 eV). Similar results can be obtained when the adsorption happen on other functional groups (imino and methyl), indicating the important role of N in Fe‐N_4_‐C structure for SMX adsorption (Figure 6f–k and Figure S22a–d: Supporting Information).

Combined with the calculated adsorption results of organic molecules, a new mechanism is proposed for the degradation process on single‐Fe‐atom materials (Figure 6l). Firstly, SMX is adsorbed onto the N in Fe‐N_4_‐C structure via the van der Waals force. Then, the single Fe atoms could activate the PMS to generate SO_4_^•−^ radicals, which are then sequently transformed into •OH and O_2_^•−^ radicals for the degradation. The dual reaction sites (N and Fe) in the single‐atom Fe‐N_4_‐C materials greatly shorten the migration distance of the active O_2_^•−^ radicals. Consequently, the in situ generated O_2_^•−^ can react with adsorbed SMX facilely, thus could obtaining enhanced ability to activate PMS for the remediation of organic pollutants.

## 3 Conclusion

In summary, we have successfully anchored single Fe atoms on porous N‐doped carbon (Fe‐N_4_‐PC) using a chemical vapor deposition (CVD) method and demonstrated their outstanding ability and stability to activate peroxymonosulfate (PMS) for the remediation of organic pollutants. The single Fe atoms are proved to be in the form of Fe‐N_4_ according to the XAFS results. During the PMS activation process, the Fe‐N_4_‐C structures are the main active sites and act dual roles: 1) the connected N in Fe‐N_4_‐C structures would enhance the adsorption of organic molecules onto the materials, 2) the single Fe atoms could activate the PMS to generate O_2_^•−^ radical, thus transforming the PMS activation pathway from non‐radical on NPC into the free radical process. These results provide deep insights of single‐atom‐based activators in the Fenton‐like reactions. The proposed single‐atom activators synthesis method and dual reaction‐site mechanism shall promote the real application of the persulfates in the environmental remediation.

## 4 Experimental Section

### Chemicals

Iron(III) acetylacetonate (98%), pyridine (C_5_H_5_N, >99.5%), sulfamethoxazole (SMX, 99%), and magnesium hydroxide were bought from Aladdin Industrial Corporation. PMS (2KHSO_5_·3KHSO_4_·K_2_SO_4_) and potassium iodide were obtained from Chemistry. DMPO and TMPO radical scavengers were purchased from Tianjin Kermel Chemistry. Methanol (MeOH), t‐butanol (TBA), furfuryl alcohol (FFA), benzoquinone (BQ), sulfuric acid and dimethyl sulfoxide (DMSO) were obtained from Tianjin Yuanli Chemistry.

### Preparation of the of Fe‐N_4_‐PC Nanocomposite

The synthesis of Fe‐N_4_‐PC with single‐atom dispersion was prepared by a two‐step process. In a typical procedure, iron(III) acetylacetonate (0.1413 g, 0.40 mmol) was first added into pyridine and stirred it for 2 h to make it evenly mixed. Then, the obtained liquid was carried by argon flow (200 sccm) into the tube furnace for pyrolysis at 800°C with a ramp rate of 5 min^−1^ for 1 h. During pyrolysis, magnesium hydroxide template (0.3499 g, 6.0 mmol) was treated as the template for deposition. After that, magnesium hydroxide template was removed by treating the material with 1 m H_2_SO_4_ solution at 45°C for 12 h. The resulting solid was washed with ethanol and deionized water for several times, followed by oven dried at 60 °C for 12 h. The obtained samples were denoted as Fe‐N_4_‐PC‐1, Fe‐N_4_‐PC‐2, and Fe‐N_4_‐PC‐3 with pyrolysis at 700, 800, and 900°C, respectively.

### Preparation of the Fe‐N_4_‐C‐2 Nanocomposite

The preparation process of Fe‐N_4_‐C‐2 catalyst was the same as that of the Fe‐N_4_‐PC‐2 catalyst except that MgO was used as the metal template. Particularly, MgO template is obtained by the decomposition of Mg(OH)_2_.

### Preparation of the Fe‐NPC Nanocomposite

For the synthesis of Fe‐PC, iron(III) acetylacetonate and Mg(OH)_2_ were dispersed in distilled water (100 mL) and stirred it for 2 h to make it evenly mixed. The solution was heated at 80°C for 12 h to obtain a solid mixture, followed by calcination at 800°C for 1 h under N_2_ atmosphere. After that, magnesium hydroxide template was removed by treating the material with 1 m H_2_SO_4_ solution at 45°C for 12 h. The resulting solid was washed with ethanol and deionized water for several times, followed by oven dried at 60 °C for 12 h.

### Synthesis of NPC Nanocomposite

For the synthesis of NPC, Mg(OH)_2_ template (0.3499 g, 6.0 mmol) was treated as the template for deposition. Pyridine was carried by argon flow (200 sccm) into the tube furnace for pyrolysis at 800°C with a ramp rate of 5 min^−1^ for 1 h. After that, magnesium hydroxide template was removed by treating the material with 1 m H_2_SO_4_ solution at 45°C for 12 h. The resulting solid was washed with ethanol and deionized water for several times, followed by oven dried at 60 °C for 12 h.

### Characterization

XRD patterns were conducted using Bruker Nonius D8 FOCUS diffractometer to determine its crystal structure equipped with Cu K*α* radiation. Related functional groups of the synthesized material were examined by FT‐IR with the model of Thermal‐Nicolet 380. Raman spectroscopy could be used to investigate the defect degree (Renishaw inVia reflex, 633 nm laser). HR‐TEM images were observed by high‐resolution transmission electron microscopy (JEM‐F200) to analyze the morphology and existence of metal. AC HAADF‐STEM characterization was conducted on JEOL JEM‐ARM200F STEM/TEM to prove the single‐atom structure. ^57^Fe Mössbauer spectra was collected MFD‐500AV‐01 at 25 °C. The concentration of elements was quantified by ICP‐MS (Optima 2100DV). The specific surface area and pore size distribution of the catalyst can be further determined by BET analysis (Bjbuilder SSA‐7000). X‐ray absorption fine structure (XAFS) and X‐ray absorption near‐edge structure spectra (XANES) were conducted at the Singapore Synchrotron Irradiation Facility (BSRF, 1W1B) to further demonstrate the existence of possible atomic structures. To determine the bonding and valence of various elements, XPS spectra was recorded on the PerkinElmer PHI 1600 X‐ray photoelectron spectroscope. The intermediates and degradation pathways of pollutants could be identified by LC‐MS spectrometry (Thermo, USA, C18 analytical column). EPR spectra of different radicals were obtained by Bruker EPR A300 spectrometer.

### Catalytic Activity Test

The catalytic degradation experiments were conducted in a batch reactor under continuously stirring (350 rpm). Sulfuric acid and sodium hydroxide was used to adjust initial solution pH of SMX solution. Typically, desired dosages of catalyst (30 mg L^−1^) and PMS (0.30 × 10^−3^ m) were added into 100 mL SMX solution. At a certain time, 1 mL solution was withdrawn and filtered by a 0.22 µm PTFE syringe filter disc, followed by the addition of 50 µL ethanol to scavenge the reaction. The residual concentration of SMX was determined via HPLC (Agilent, U3000, XDB‐C18 column). The mobile phase consists of 40% 0.1% acetic acid and 60% acetonitrile mixture with a flow rate of 1 mL min^−1^.

### Stability and Quenching Tests

For the stability test, the used catalyst was recycled and further washed with water and alcohol to remove some adsorbed impurities. For the quenching of various free radicals, methanol (MeOH, 0.5 m), tert‐butyl alcohol (TBA, 0.5 m), p‐benzoquinone (BQ, 12 × 10^−3^ m), furfuryl alcohol (FFA, 12 × 10^−3^ m), potassium iodide (KI, 12 × 10^−3^ m) and dimethyl sulfoxide (DMSO, 12 × 10^−3^ m) were used as quenchers for different radicals. In addition, the concentration of leached iron was quantified by inductively coupled plasma mass spectrometry (ICP‐MS).

## Conflict of Interest

The authors declare no conflict of interest.

## Supporting information

Supporting Information

## Data Availability

The data that support the findings of this study are available from the corresponding author upon reasonable request.
