# Supplementary material for: Facile Synthesis of Atomic Fe‐N‐C Materials and Dual Roles Investigation of Fe‐N4 Sites in Fenton‐Like Reactions
Source: Adv Sci (Weinh). 2021 Oct 12;8(22):2101824. doi: 10.1002/advs.202101824 (PMC8596112; doi:10.1002/advs.202101824)
Supplement: Supplementary file 1 — Supporting Information [file ADVS-8-2101824-s001.pdf]

© 2021 Wiley-VCH GmbH

ADVANCED SCIENCE
Open Access

Supporting Information

for *Adv. Sci.*, DOI: 10.1002/advs.202101824

Facile Synthesis of Atomic Fe-N-C Materials and Dual Roles Investigation of Fe-N_4 sites in Fenton-like Reactions

*Jun Wang, Bin L, Yang Li, Xiaobin Fan, Fengbao Zhang, Guoliang Zhang, and Wenchao Peng**

Supporting Information

Facile Synthesis of Atomic Fe-N-C Materials and Dual Roles Investigation of Fe-N_4 sites in Fenton-like Reactions

*Jun Wang, Bin L, Yang Li, Xiaobin Fan, Fengbao Zhang, Guoliang Zhang, and Wenchao Peng**

Mr. J. Wang, Mr. L. Wu, Mr. B. Li, Dr. Y. Li, Prof. X. Fan, Prof. F. Zhang, Prof. G. Zhang, Prof. W. Peng.
Department of Chemical Engineering, Tianjin University, Tianjin 300350, China

Dr. Y. Li, Prof. X. Fan, Prof. W. Peng.
Chemistry and Chemical Engineering Guangdong Laboratory, Shantou, 515031, China

E-mail: wenchao.peng@tju.edu.cn

Contents

1. Experimental Procedures

Text S1 The effect of Cl^-, $H_2PO_4^-$, NO_3^-, and HCO_3^- in ROS process.

Text S2 Generation of various radicals $^1O_2$ radical.

Text S3 Computational Framework.

2. Supplementary Figures S1-S22

Figure S1. N_2 adsorption-desorption isotherms of different catalyst. (a) NPC. (b) Fe-N_4-PC-1. (c) Fe-N_4-PC-2. (d) Fe-N_4-PC-3. (e) Fe-N_4-C-2. (f) Fe-PC (inset was the pore size distribution).

Figure S2. (a) XRD spectra of Fe-N_4-C-2, Fe-N_4-PC-2, Fe-N_4-C-2 Fresh (Before acid leaching), Fe-N_4-PC-2 Fresh (Before acid leaching) catalysts. (b) XRD spectra of NPC, Fe-N_4-C-1, Fe-N_4-C-2 and Fe-N_4-C-3 catalysts.

Figure S3. Raman spectra of Fe-PC, NPC, Fe-N_4-PC-1, Fe-N_4-PC-2 and Fe-N_4-C-2 and Fe-N_4-PC-3 catalysts. All prepared catalysts show similar intensity ratio of the D-band/G-band (I_D /I_G), suggesting similar disorientated degree of graphene.

Figure S4. (a-d) Aberration-corrected HAADF-STEM images of Fe-N_4-PC-2

Figure S5. The corresponding EXAFS fitting curves of Fe-N_4-PC-2 at k space.

Figure S6 Corresponding EXAFS fitting curves of Fe, FeO and Fe_2O_3 samples. (a) Fe foil at k space. (b) Fe foil at R space. (a) FeO at k space. (b) FeO at R space. (a) Fe_2O_3 at k space. (b) Fe_2O_3 at R space.

Figure S7. XPS survey spectra of NPC, Fe-N_4-PC-1, Fe-N_4-PC-2, Fe-N_4-C-2 and Fe-N_4-PC-3 catalysts.

Figure S8. N 1s XPS spectra of (a) NPC, (b) Fe-N_4-PC-1, (c) Fe-N_4-C-2 and (d) Fe-N_4-PC-3 catalysts.

Figure S9. The 5 binding states of N in Fe-N_4-PC-2 single-atom catalyst.

Figure S10. N_2 adsorption-desorption isotherms of (a) $Mg(OH)_2$ and (b) MgO template (inset was the pore size distribution).

Figure S11. (a) Effects of as-prepared Fe-N_4 SAs-NPC-2 sample. Reaction condition: [SMX] = 10 ppm, [PMS] = 0.30 mM, T = 298 K, initial solution pH = 6.0. (b) Effects of PMS concentration. Reaction condition: SMX concentration = 10 ppm, PMS concentration = 0.30 mM, Catalyst dosage = 30 mg/L, Reaction temperature = 298 K and initial pH value = 6.0.

Figure S12. Measurement of pH_{ZPC} for (a) NPC and (b) Fe-N_4-PC-2 catalysts.

Figure S13. Effects of (a) Cl^-, (b) $H_2PO_4^-$, (c) NO_3^-, and (d) HCO_3^- on the SMX degradation in Fe-N_4-PC-2/PMS system. Reaction condition: SMX concentration = 10 ppm, PMS concentration = 0.30 mM, Catalyst dosage = 30 mg/L, Reaction temperature = 298 K and initial pH value = 6.0.

Figure S14. Degradation performance of different organic contaminants with Fe-N_4-PC-2 as catalyst. Reaction condition: pollution concentration = 10 ppm, PMS concentration = 0.30 mM, Catalyst dosage = 30 mg/L, Reaction temperature = 298 K and initial pH value = 6.0.

Figure S15. Degradation performance of different oxidants with Fe-N_4-PC-2 as catalyst.

Reaction condition: SMX concentration = 10 ppm, PMS concentration = 0.30 mM, Catalyst dosage = 30 mg/L, Reaction temperature = 298 K and initial pH value = 6.0.

Figure S16. Oxidation state of Fe-N_4-C_x structure in Fe-N-PC-2/PMS system.

Figure S17. Annealing treatment after 4th cycles in Fe-N-PC-2/PMS system.

Reaction condition: SMX concentration = 10 ppm, PMS concentration = 0.30 mM, Catalyst

dosage = 30 mg/L, Reaction temperature = 298 K and initial pH value = 6.0.

Figure S18. Decline of PMSO concentration and production of $PMSO_2$ in the Fe-N_4-PC-2/PMS system. Reaction condition: PMSO concentration = 20 uM, PMS concentration = 200 uM, Catalyst dosage = 30 mg/L, Reaction temperature = 298 K and initial pH value = 6.0.

Figure S19. Mass spectrum of degradation products for SMX degradation in Fe-N_4-PC-2/PMS system.

Figure S20. Proposed SMX degradation pathways over Fe-N_4-PC/PMS system.

Figure S21. Optimized configurations of PMS adsorbed on different catalysts (top view). (a) Carbon. (b) Graphitic N. (c) Pyrrolic N. (d) Pyridine N. (e) Fe-foil. (f) FeO. (g) Fe_2O_3. (h) Fe-N_4-PC-2 catalyst.

Figure S22. Local adsorption configurations of imino (-NH-) on (a) Pyridinic N and (b) Oxide N structure. Local adsorption configurations of methyl (-CH_3) on (c) Pyridinic N and (d) Oxide N structure.

3. Supplementary Tables S1-S7

Table S1. BET specific surface area (SSA), total pore volume and pore width of Fe-N_4-PC-2 and other samples.

Table S2. Best fitting EXAFS data for Fe-N_4-PC-2 and reference samples.

Table S3 Mossbauer parameters and relative absorption area obtained for each component from the fitting of the experimental spectrum recorded at room temperature.

Table S4. The ratios of different N to total N content in samples.

Table S5 The reaction rate constants of EtOH, TBA, FFA and BQ with different ROSs.

Table S6. The lifespan of singlet oxygen ($^1O_2$) in different solutions.

Table S7. Chemical components of Fe-N-PC-2 in Fe-N-PC-2/PMS system.

Table S8. The catalytic performance comparison of recently reported Fenton-like catalysts for PMS activation and pollutants degradation.

Table S9. Possible intermediate of SMX degradation.

Table S10. Theoretical computations of PMS adsorption onto different models.

Table S11. Theoretical computations of -NH_2, -NH and -CH_3 adsorption onto different models.

4. References 1-15

Text S1 The effect of Cl^-, $H_2PO_4^-$， NO_3^-， and HCO_3^- in ROS process.

1. The effect of Cl^- ion in ROS process.

$$Cl^- + {}^{\bullet}OH \rightarrow ClHO^{\bullet-} \quad (S1)$$

$$ClHO^{\bullet-} + H^+ \rightarrow Cl^{\bullet} + H_2O \quad (S2)$$

$$SO_4^{\bullet-} + Cl^- \rightarrow Cl^{\bullet} + SO_4^{2-} \quad (S3)$$

$$Cl^- + HSO_5^- \rightarrow SO_4^{2-} + HOCl \quad (S4)$$

$$2Cl^- + HSO_5^- + H^+ \rightarrow SO_4^{2-} + Cl_2 + H_2O \quad (S5)$$

2. The effect of $H_2PO_4^-$ ion in ROS process.

$$H_2PO_4^- + SO_4^{\bullet-} \rightarrow SO_4^{2-} + H_2PO_4^{\bullet} \quad (S6)$$

$$H_2PO_4^- + {}^{\bullet}OH \rightarrow OH^- + H_2PO_4^{\bullet} \quad (S7)$$

3. The effect of NO_3^- ion in ROS process.

$${}^{\bullet}OH + NO_3^- \rightarrow OH^- + NO_3^{\bullet} \quad (S8)$$

$$SO_4^{\bullet-} + NO_3^- \rightarrow NO_3^{\bullet} + SO_4^{2-} \quad (S9)$$

$$NO_3^{\bullet} + H_2O + e_{aq}^- \rightarrow NO_2^{\bullet} + 2OH^- \quad (S10)$$

4. The effect of HCO_3^- ion in ROS process.

Adding HCO_3^- ion with buffering capacity can lead to a slight decrease in pH, which facilitates the production of superoxide radicals, thus improving the degradation performance.

$$H^+ + O_2^{\bullet-} \rightarrow HO_2^{\bullet} \quad (S11)$$

$$2O_2^{\bullet-} + 2H_2O \rightarrow {}^1O_2 + H_2O_2 + OH^- \quad (S12)$$

Text S2 Generation of various radicals $^1O_2$ radical.

$HSO_5^- + SO_5^{2-} \rightarrow HSO_4^- + SO_4^{2-} + {}^1O_2$ (S1)

$HSO_5^- + H_2O \rightarrow HSO_4^- + H_2O_2$ (S2)

$H_2O_2 \rightarrow H^+ + HO_2^{\bullet-}$ (S3)

$HO_2^{\bullet-} \rightarrow H^+ + O_2^{\bullet-}$ (S4)

$\bullet OH + O_2^{\bullet-} \rightarrow {}^1O_2 + OH^-$ (S5)

$2O_2^{\bullet-} + 2H_2O \rightarrow {}^1O_2 + H_2O_2 + 2OH^-$ (S6)

Text S3 Computational Framework.

The models were computed with density functional theory (DFT) using projected augment wave method as implemented in the Vienna ab initio Simulation Package (VASP) code. The generalized gradient approximation (GGA) of Perdew-Burke-Ernzerhof (PBE) is used for the exchange-correlation potential. Plane-wave basis set was used with an energy cutoff of 500 eV. The convergence criterion for electronic structure iteration was set to be 1×10^{-4} eV and structural optimization would be terminated until all forces were smaller than 0.05eV/Å. The (3 × 3) and (2 × 2) supercells with four atomic layers were cleaved to mimic Fe (0001), FeO (100) and Fe_2O_3 (100) surfaces, respectively. The vacuum layer was about 13 Å. The upper two atomic layers of slabs and adsorbates were free to move in all directions, while the bottom two layers were fixed at the ground-state bulk positions. The surface Brillouin-zone of Fe (0001) and FeO (100) surfaces were sampled using the k-meshes of 6×6×1 and 4×4×1, respectively. Note that Fe and Fe_2O_3 are an antiferromagnetic oxide; the antiferromagnetic calculations of PMS adsorption on Fe and Fe_2O_3 (100) surface with spin alignment planes on the (111) and (100) planes were considered. The adsorption of PMS on graphitic N-doped carbon and FeN_4-carbon was simulated using a (8 × 8) supercell with a vacuum layer of 20 Å. A k-mesh of 2×2×1 was used for the sampling of the Brillouin-zone. The adsorption of SMX on pyrrolic, pyridinic, and graphitic N-doped graphene was carried out in slab models in dimensions of 30.76 Å×17.11 Å×20 Å, 17.29 Å×28.11 Å×20 Å, and 19.76 Å×19.76 Å ×20 Å with k-meshes of 1×2×1, 2×1×1, and 2×2×1 to sample the first Brillouin-zone, respectively. The atoms were relaxed fully until the force acting on each atom

was less than 0.02 eV/Å. van der Waals (vdW) interaction was taken into account at the DFT-D2 level as proposed by Grimme. The adsorption energy (E_{ads}) of PMS onto different substrates can be defined as:

$$E_{ads} = E_{total} - E_{substrate} - E_{molecule} \qquad (4)$$

Where E_{total}, $E_{substrate}$ and $E_{molecule}$ denote the total energy of substrate with adsorbate, substrate, and free molecule, respectively.

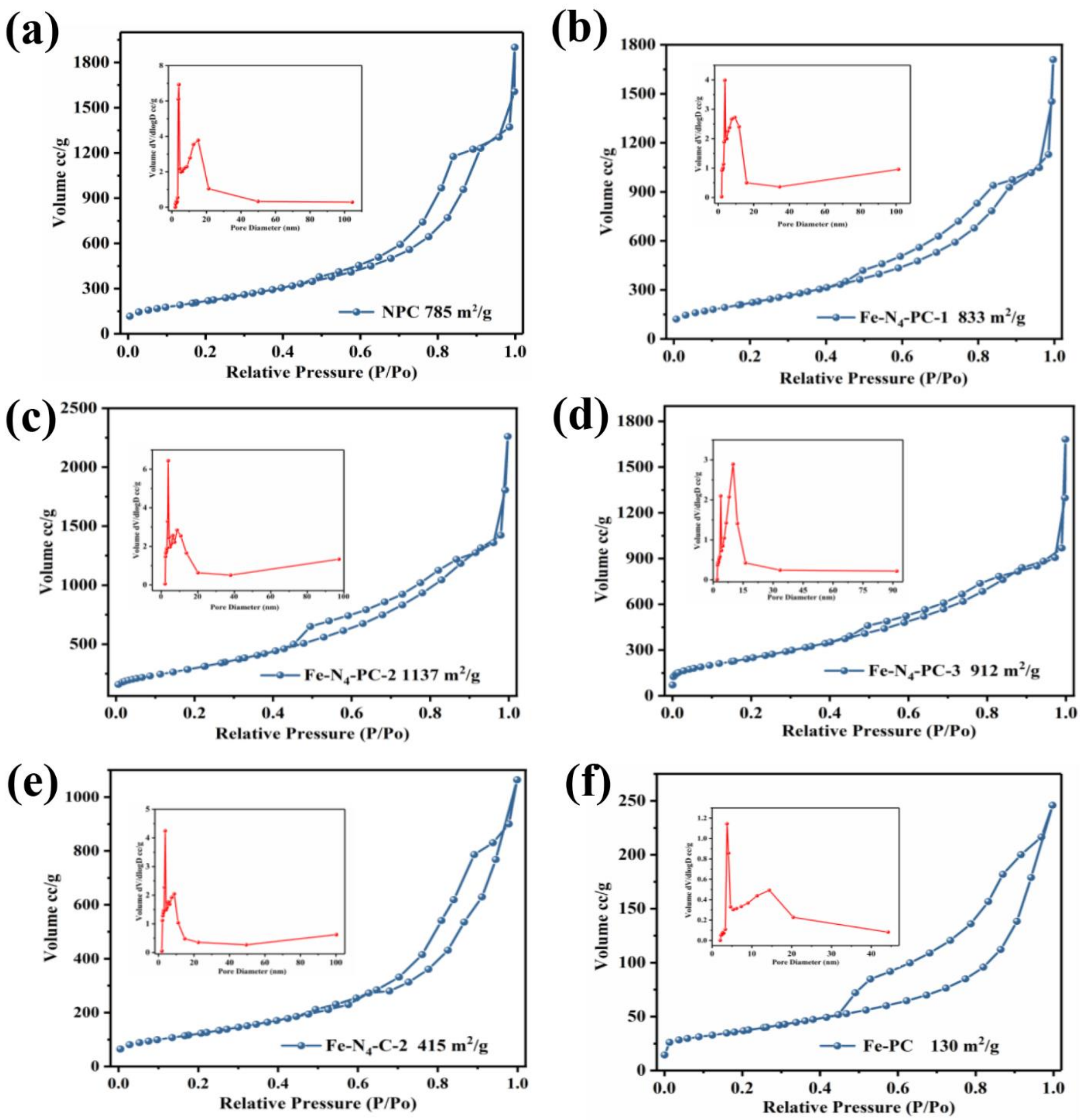

Figure S1. N_2 adsorption-desorption isotherms of different catalyst. (a) NPC. (b) Fe-N_4-PC-1. (c) Fe-N_4-PC-2. (d) Fe-N_4-PC-3. (e) Fe-N_4-C-2. (f) Fe-PC (inset was the pore size distribution).

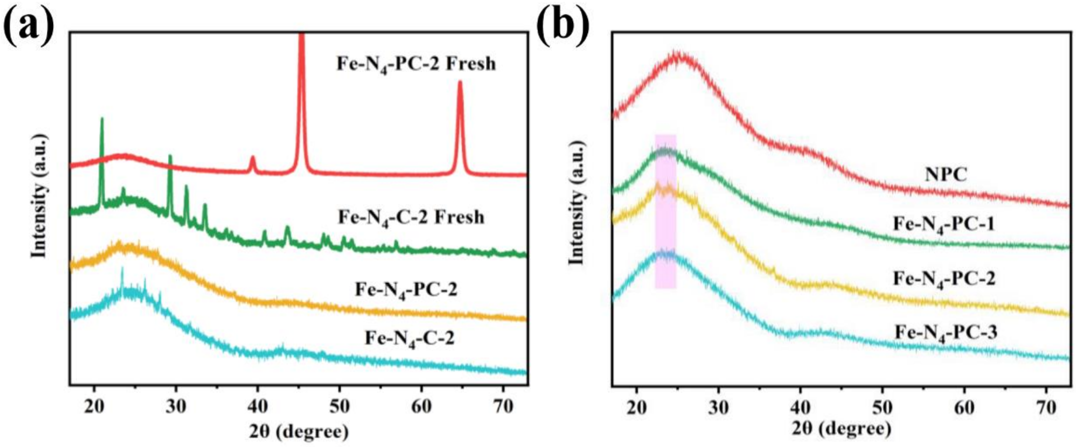

Figure S2. (a) XRD spectra of Fe-N_4-C-2, Fe-N_4-PC-2, Fe-N_4-C-2 Fresh (Before acid leaching), Fe-N_4-PC-2 Fresh (Before acid leaching) catalysts. (b) XRD spectra of NPC, Fe-N_4-C-1, Fe-N_4-C-2 and Fe-N_4-C-3 catalysts.

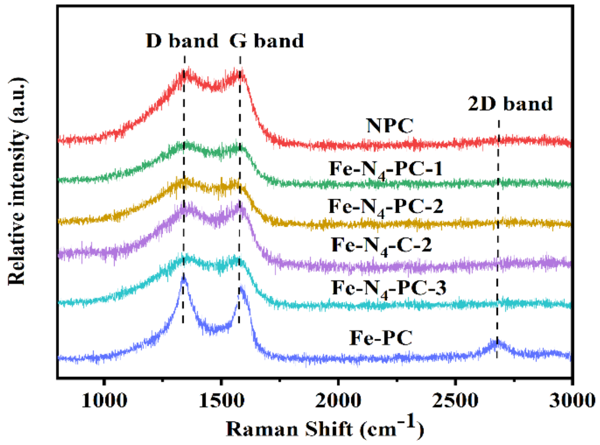

Figure S3. Raman spectra of Fe-PC, NPC, Fe-N_4-PC-1, Fe-N_4-PC-2 and Fe-N_4-C-2 and Fe-N_4-PC-3 catalysts. All prepared catalysts show similar intensity ratio of the D-band/G-band (I_D /I_G), suggesting similar disorientated degree of graphene.

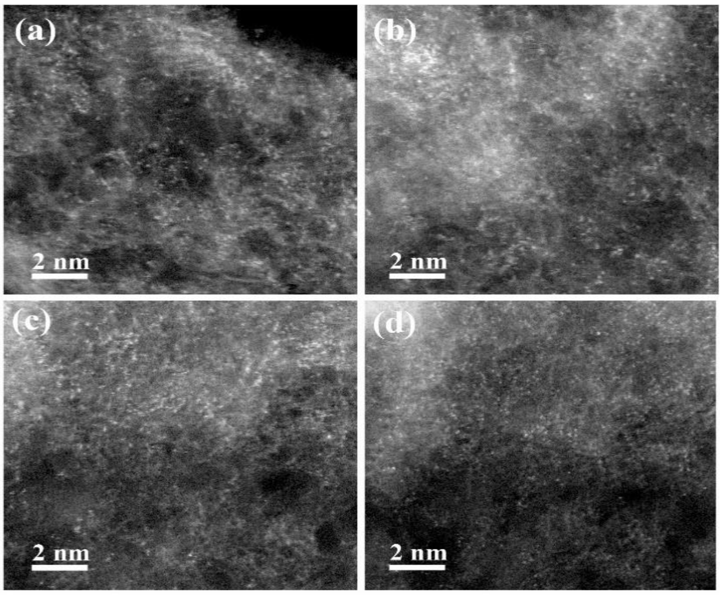

Figure S4. (a-d) Aberration-corrected HAADF-STEM images of Fe-N_4-PC-2.

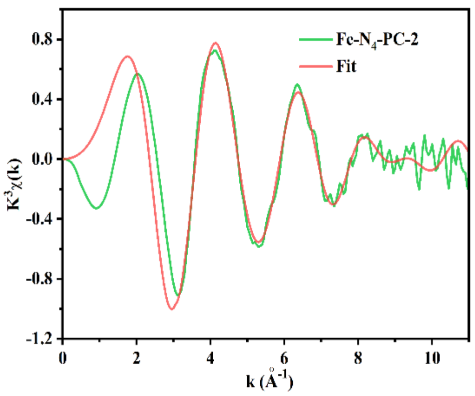

Figure S5. The corresponding EXAFS fitting curves of Fe-N_4-PC-2 at k space.

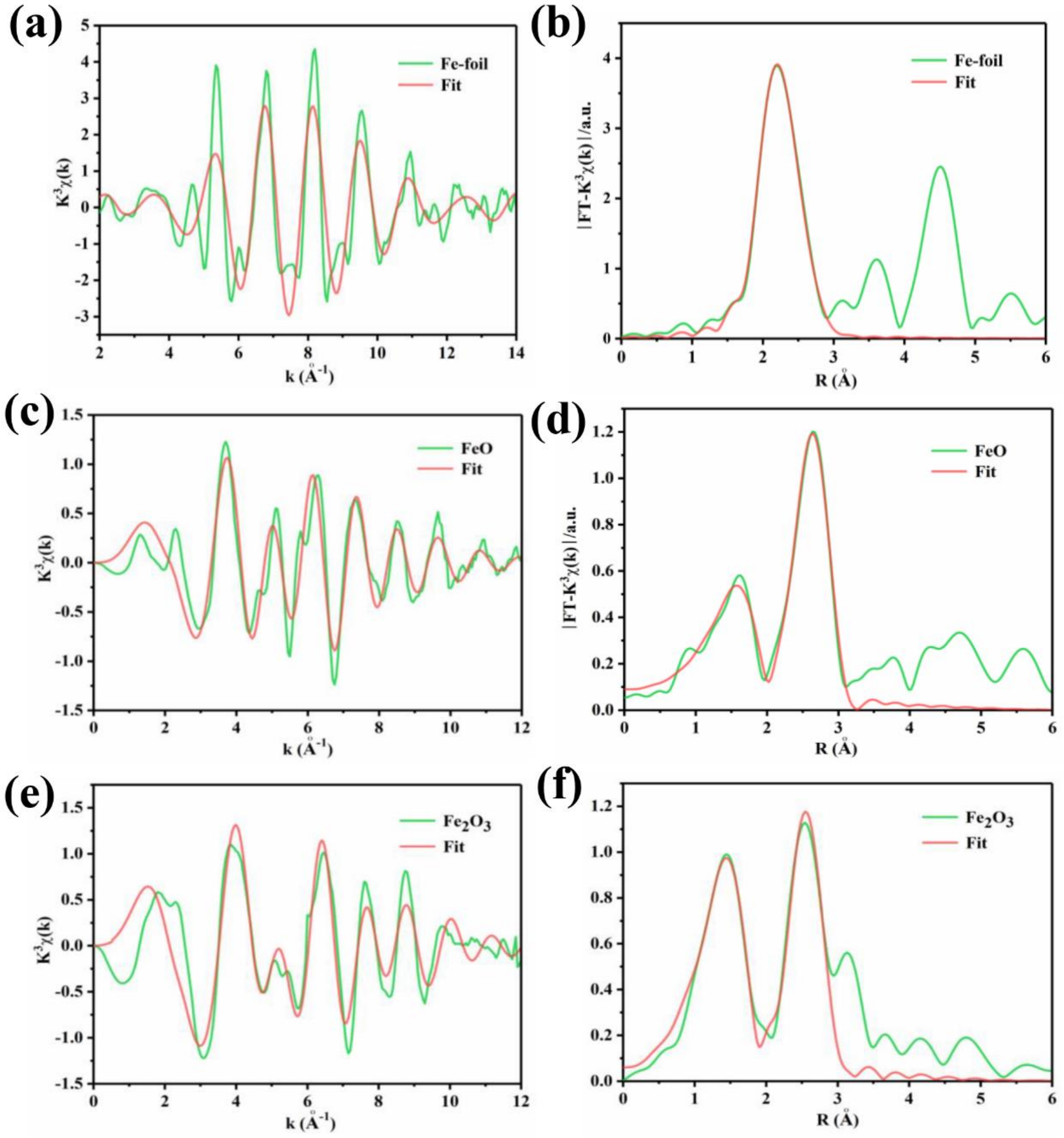

Figure S6 Corresponding EXAFS fitting curves of Fe, FeO and Fe_2O_3 samples. (a) Fe foil at k space. (b) Fe foil at R space. (a) FeO at k space. (b) FeO at R space. (a) Fe_2O_3 at k space. (b) Fe_2O_3 at R space.

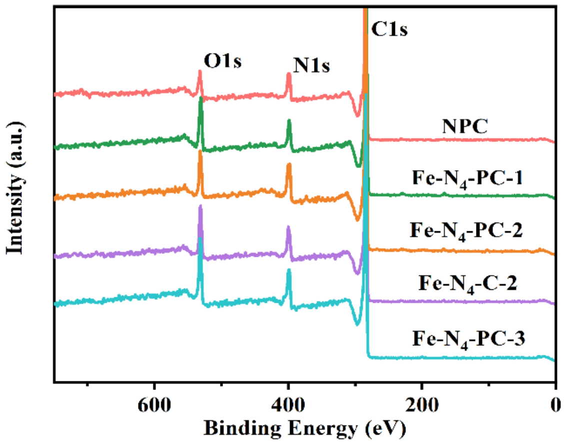

Figure S7. XPS survey spectra of NPC, Fe-N_4-PC-1, Fe-N_4-PC-2, Fe-N_4-C-2 and Fe-N_4-PC-3 catalysts.

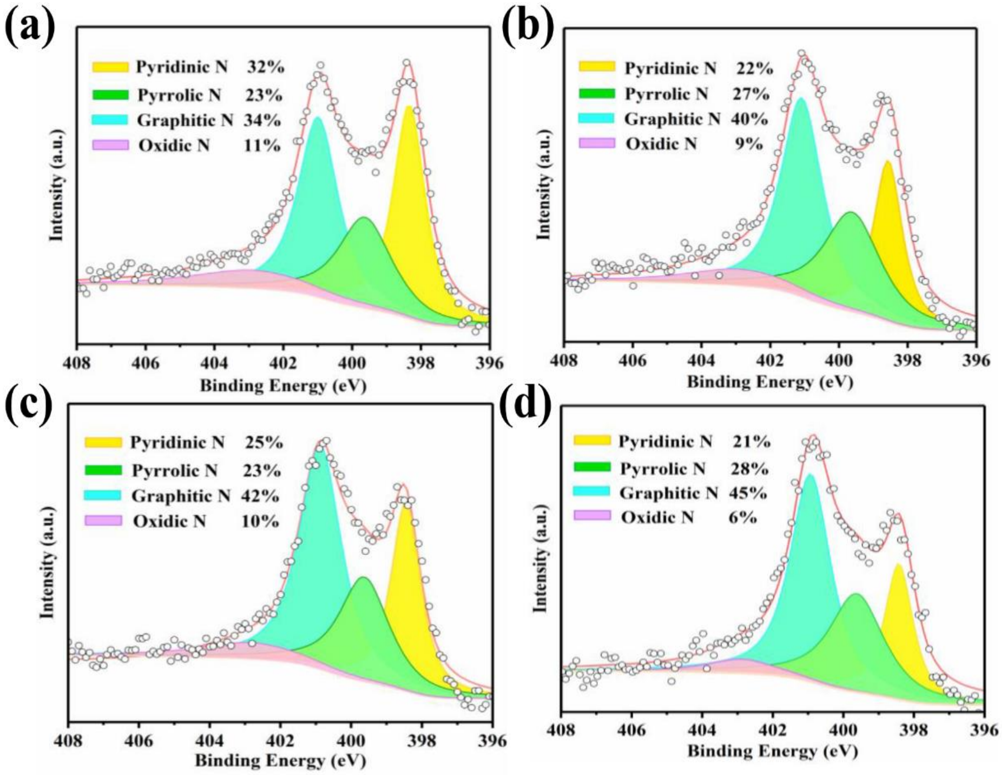

Figure S8. N 1s XPS spectra of (a) NPC, (b) Fe-N_4-PC-1, (c) Fe-N_4-C-2 and (d) Fe-N_4-PC-3 catalysts.

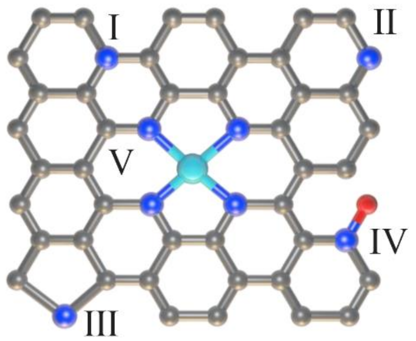

Figure S9. The 5 binding states of N in Fe-N_4-PC-2 single-atom catalyst.

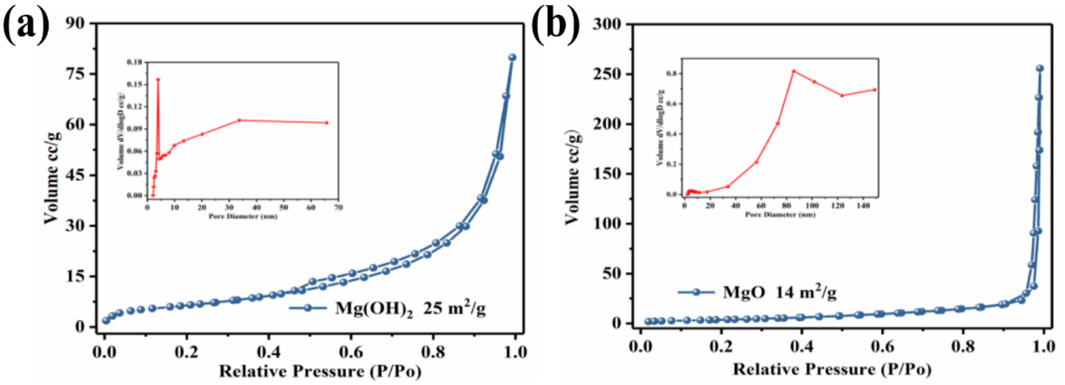

Figure S10. N_2 adsorption-desorption isotherms of (a) $Mg(OH)_2$ and (b) MgO template (inset was the pore size distribution).

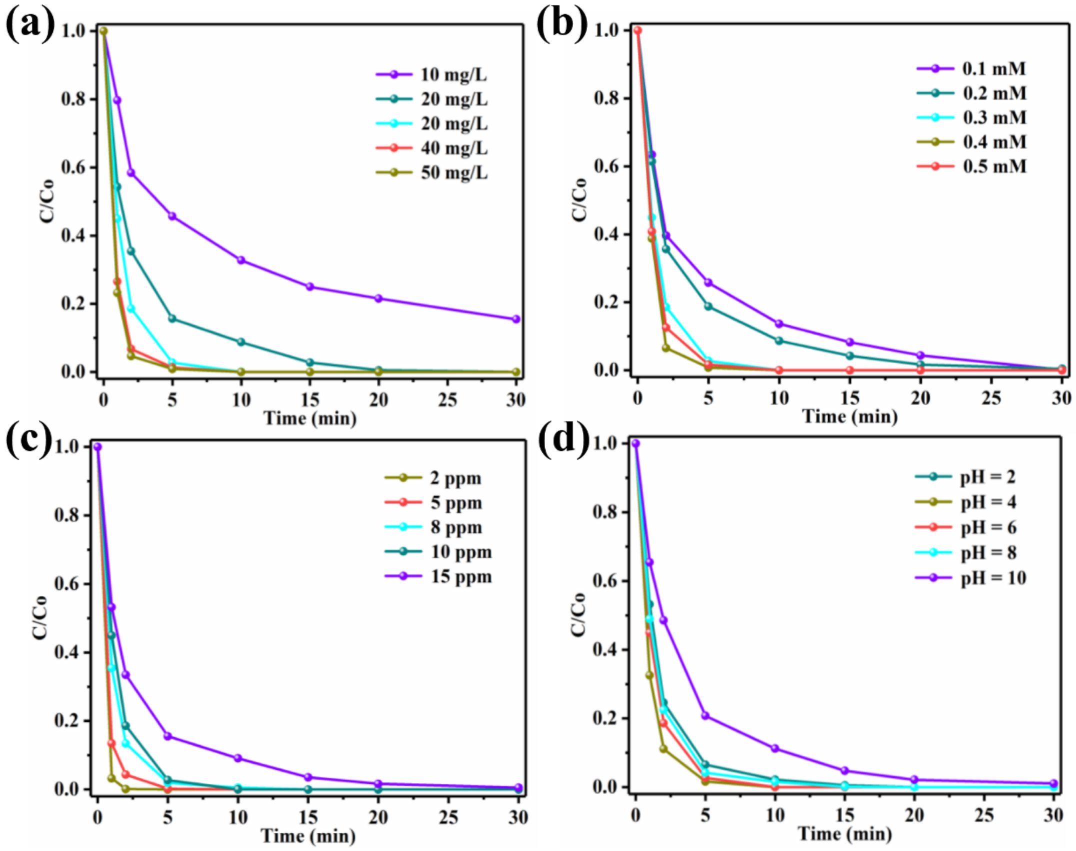

Figure S11. (a) Effects of as-prepared Fe-N_4 SAs-NPC-2 sample. Reaction condition: [SMX] = 10 ppm, [PMS] = 0.30 mM, T = 298 K, initial solution pH = 6.0. (b) Effects of PMS concentration. Reaction condition: SMX concentration = 10 ppm, PMS concentration = 0.30 mM, Catalyst dosage = 30 mg/L, Reaction temperature = 298 K and initial pH value = 6.0.

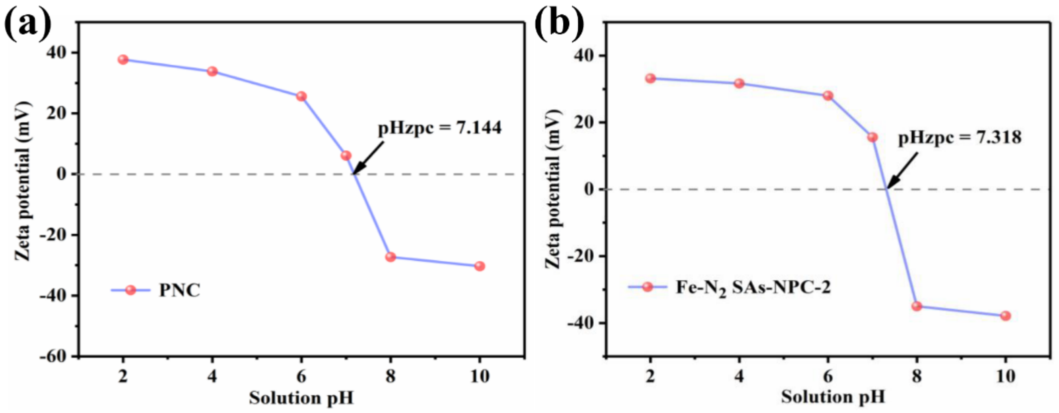

Figure S12. Measurement of pH_{ZPC} for (a) NPC and (b) Fe-N_4-PC-2 catalysts.

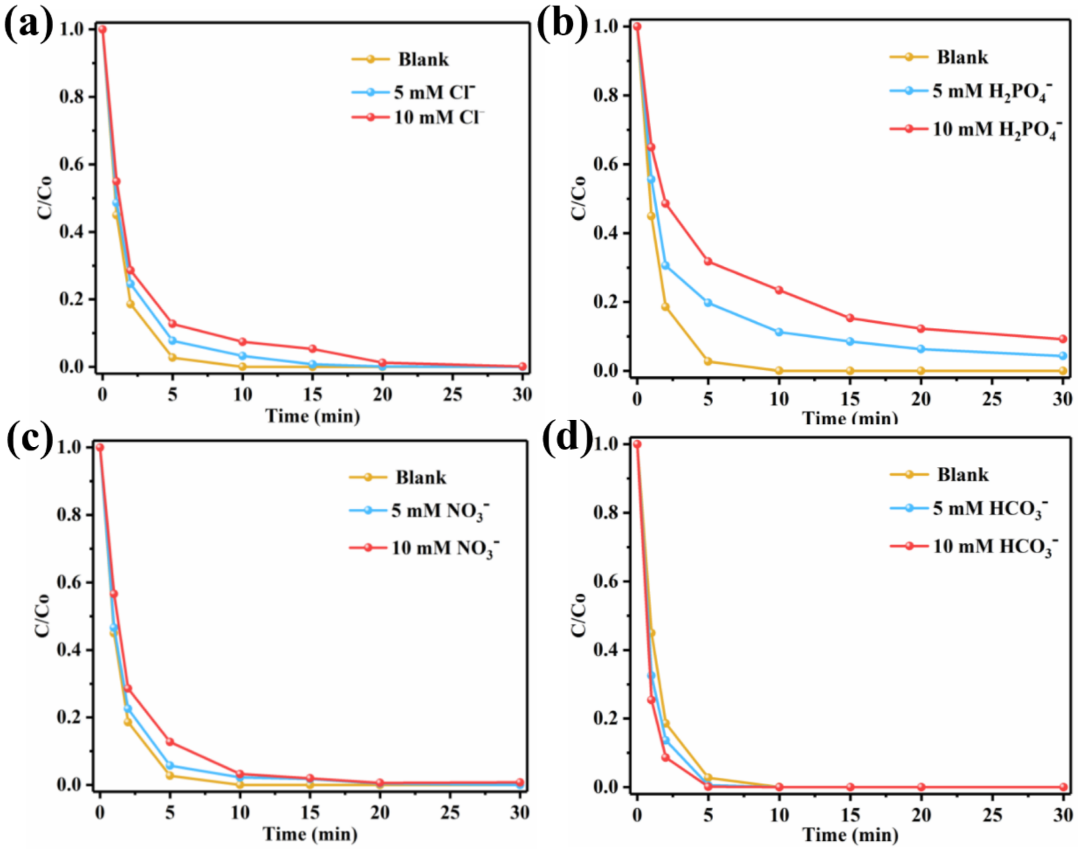

Figure S13. Effects of (a) Cl^-, (b) $H_2PO_4^-$, (c) NO_3^-, and (d) HCO_3^- on the SMX degradation in Fe-N_4-PC-2/PMS system. Reaction condition: SMX concentration = 10 ppm, PMS concentration = 0.30 mM, Catalyst dosage = 30 mg/L, Reaction temperature = 298 K and initial pH value = 6.0.

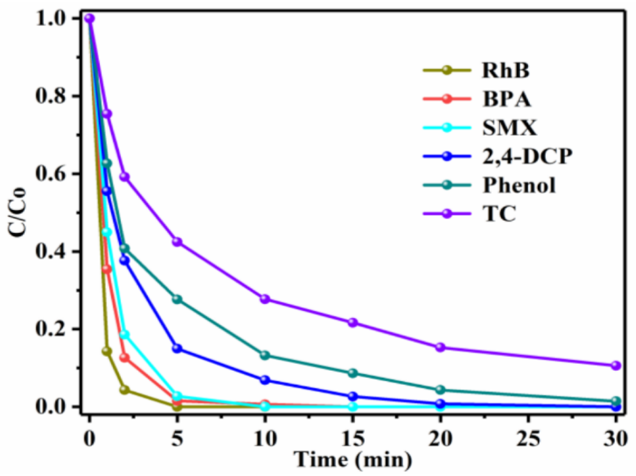

Figure S14. Degradation performance of different organic contaminants with Fe-N_4-PC-2 as catalyst. Reaction condition: pollution concentration = 10 ppm, PMS concentration = 0.30 mM, Catalyst dosage = 30 mg/L, Reaction temperature = 298 K and initial pH value = 6.0.

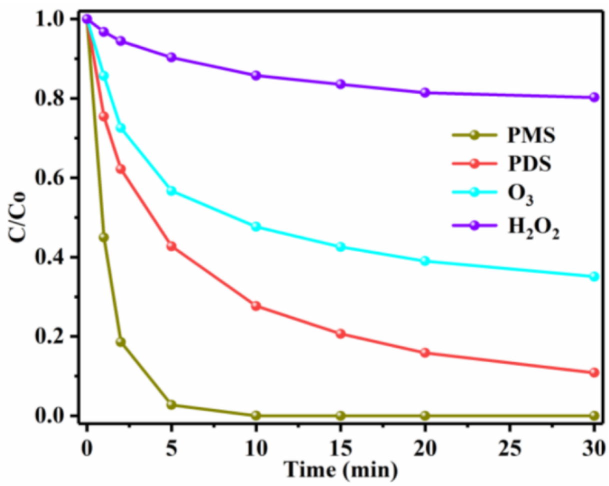

Figure S15. Degradation performance of different oxidants with Fe-N_4-PC-2 as catalyst. Reaction condition: SMX concentration = 10 ppm, PMS concentration = 0.30 mM, Catalyst dosage = 30 mg/L, Reaction temperature = 298 K and initial pH value = 6.0.

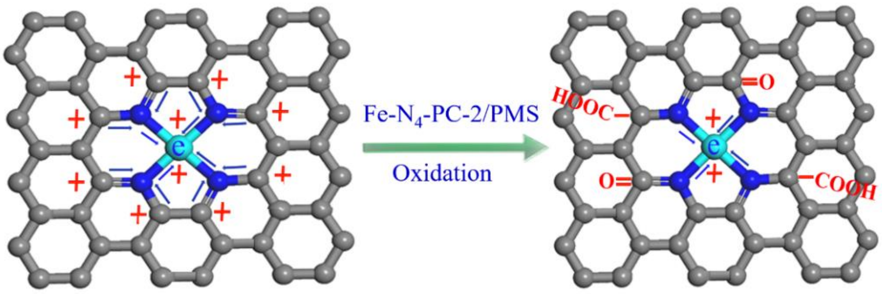

Figure S16. Oxidation state of Fe-N_4-C_x structure in Fe-N-PC-2/PMS system.

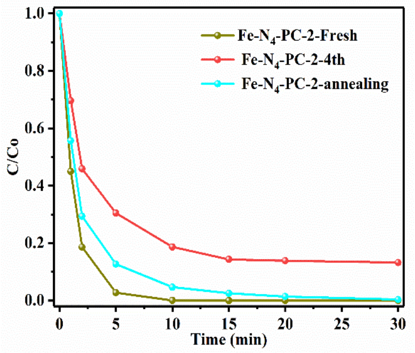

Figure S17. Annealing treatment after 4th cycles in Fe-N-PC-2/PMS system. Reaction condition: SMX concentration = 10 ppm, PMS concentration = 0.30 mM, Catalyst dosage = 30 mg/L, Reaction temperature = 298 K and initial pH value = 6.0.

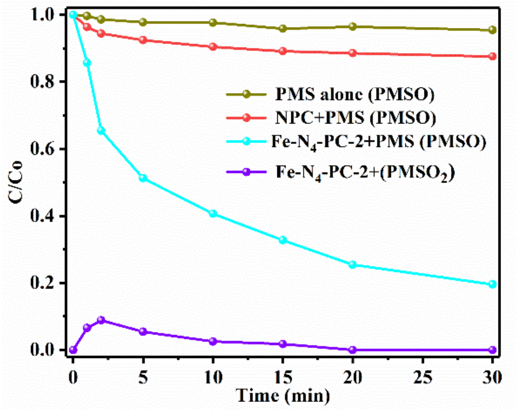

Figure S18. Decline of PMSO concentration and production of $PMSO_2$ in the Fe-N_4-PC-2/PMS system. Reaction condition: PMSO concentration = 20 uM, PMS concentration = 200 uM, Catalyst dosage = 30 mg/L, Reaction temperature = 298 K and initial pH value = 6.0.

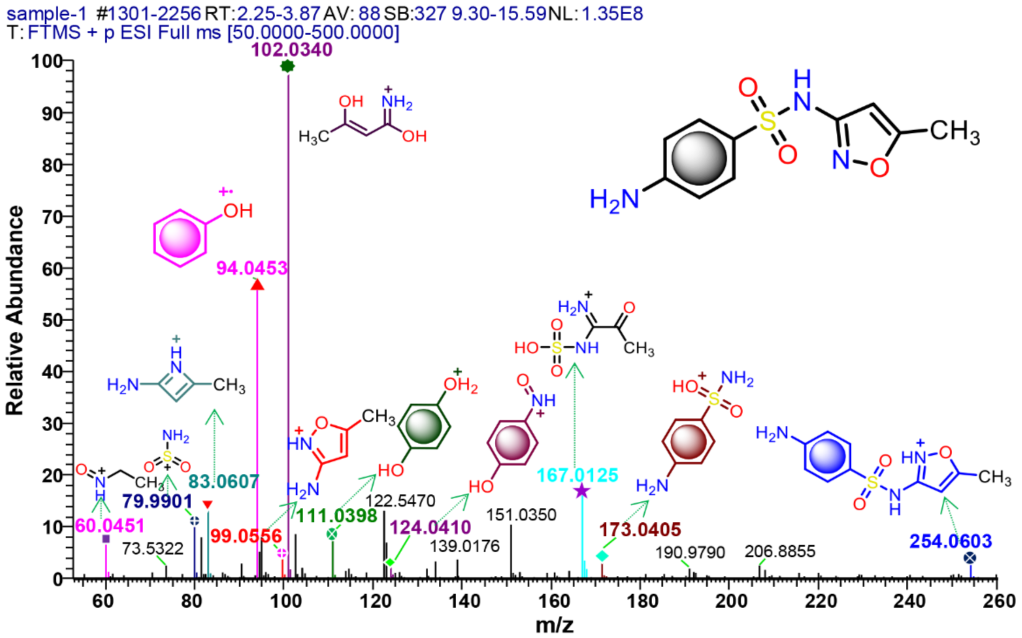

Figure S19. Mass spectrum of degradation products for SMX degradation in Fe-N_4-PC-2/PMS system.

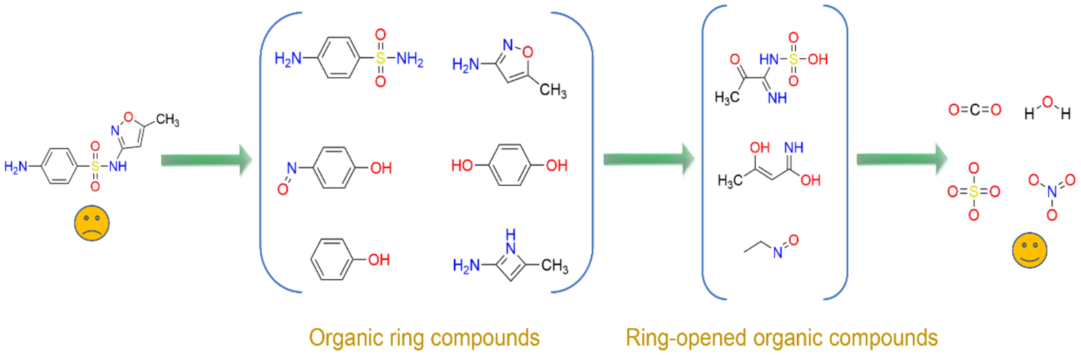

Figure S20. Proposed SMX degradation pathways over Fe-N_4-PC/PMS system.

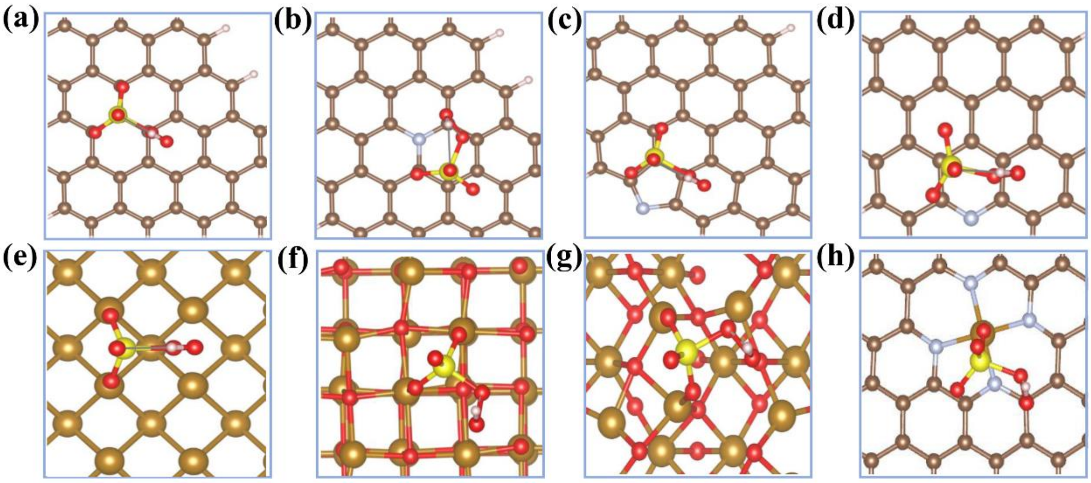

Figure S21. Optimized configurations of PMS adsorbed on different catalysts (top view). (a) Carbon. (b) Graphitic N. (c) Pyrrolic N. (d) Pyridine N. (e) Fe-foil. (f) FeO. (g) Fe_2O_3. (h) Fe-N_4-PC-2 catalyst.

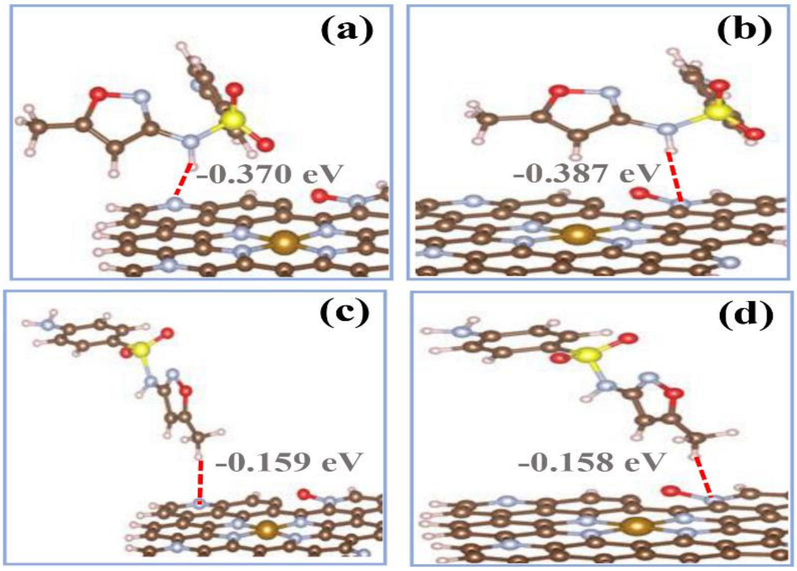

Figure S22. Local adsorption configurations of imino (-NH-) on (a) Pyridinic N and (b) Oxide N structure. Local adsorption configurations of methyl (-CH_3) on (c) Pyridinic N and (d) Oxide N structure.

Table S1. BET specific surface area (SSA), total pore volume and pore width of Fe-N_4-PC-2 and other samples.

Samples	BET specific surface area (m^2/g)	Total pore Volume (cm^3/g)
NPC	785	2.1547
Fe-N_4-PC-1	833	2.3678
Fe-N_4-PC-2	1137	3.4963
Fe-N_4-C-2	912	2.5198
Fe-N_4-PC-3	415	1.0465
Fe-PC	130	0.3801
$Mg(OH)_2$	25	0.3123
MgO	14	0.3957

Table S2. Best fitting EXAFS data for Fe-N_4-PC-2 and reference samples.

	Path	N	ΔE(eV)	100xR(Å)	10^3xσ^2(Å^2)	R-factor
Fe foil	Fe-Fe1	8	5.26(1.28)	246.2(0.6)	4.50(0.82)	0.008
	Fe-Fe2	6	5.26(1.28)	284.6(0.6)	4.50(0.82)	
FeO	Fe-O	6	-3.23(1.11)	215.7(1.8)	8.91(2.14)	0.007
	Fe-Fe	12	-4.11(1.10)	305.5(1.0)	8.74(1.02)	
Fe_2O_3	Fe-O	6	7.51(3.22)	196.1(2.5)	10.95(2.12)	0.008
	Fe-Fe	6	0.96(2.31)	298.3(1.9)	8.85(1.72)	
Sample	Fe-N	4.23(0.94)	-3.34(0.46)	199.6(1.6)	4.18(0.32)	0.001

Table S3 Mossbauer parameters and relative absorption area obtained for each component from the fitting of the experimental spectrum recorded at room temperature.

Samples	Isomer shift (IS, mm/s)	Quadrupole splitting (QS, mm/s)	Linewidth (LW, mm/s)	Area (%)	Assignment
D1	0.36	2.73	0.68	84	Distorted Fe^{II}–N_4-C intermediate spin.[22-24]
D2	0.34	1.38	0.57	16	Fe^{II}–N_4, high spin.[25, 26]
		χ^2=0.536			

Table S4. The ratios of different N to total N content in samples.

Samples	N atomic Level (%)	Pyridinic N (%)	Pyrrolic N (%)	Graphitic N (%)	N oxides (%)
NPC	5.47	32	23	34	11
Fe-N_4-PC-1	6.46	22	27	40	9
Fe-N_4-PC-2	6.85	22	15	45	18
Fe-N_4-C-2	6.13	25	23	42	10
Fe-N_4-PC-3	6.82	21	28	45	6

Table S5 The reaction rate constants of EtOH, TBA, FFA and BQ with different ROSs.

Quenchers	Reaction rate constants (M^{-1} • S^{-1})			
	$^{\bullet}OH$	$SO_4^{\bullet-}$	$^1O_2$	$O_2^{\bullet-}$
EtOH	1.9×10^9	$(1.6\text{-}7.7)\times10^7$	3.8×10^3	$<1.0\times10^3$
TBA	$(3.8\text{-}7.6)\times10^8$	$(4.0\text{-}8.1)\times10^5$	$<1.0\times10^4$	$<1.0\times10^3$
FFA	1.2×10^4	2.8×10^3	6.6×10^7	$<1.0\times10^3$
BQ	1.2×10^6	1.0×10^8	3.8×10^7	$(0.9\text{-}1.0)\times10^9$

Table S6. The lifespan of singlet oxygen ($^1O_2$) in different solutions.

Solution	H_2O	Ethanol	Cyclohexane	Benzene	Acetone	D_2O
Lifespan (μs)	2	10	16	24	25	32

Table S7. Chemical components of Fe-N-PC-2 in Fe-N-PC-2/PMS system.

Samples	C, at%	N, at%	O, at%	Fe, at%
Fe-N_4-PC-2 (Fresh)	84.12	7.95	7.71	0.22
Fe-N_4-PC-2 (4th)	78.58	6.68	14.23	0.20
Fe-N_4-PC-2 (annealing)	83.49	6.54	9.87	0.19

Table S8. The catalytic performance comparison of recently reported Fenton-like catalysts for PMS activation and pollutants degradation.

Catalysts (loading, g/L)	Pollutants ppm	PMS mM	Removal efficiency	Ref.
Fe-N_4-PC-2 (0.03)	SMX (10 ppm)	0.30	100% (15 min)	This work
Cu_1Co_1LDH (0.06)	SMX (10 ppm)	0.24	100% (30 min)	[1]
CoO@mpgCN (0.1)	SMX (5 ppm)	10	100% (15 min)	[2]
0.2CoAlLDH@CoS_x (0.1)	SMX (10 ppm)	0.3	100% (6 min)	[3]
$FeVO_4$/VL (0.5)	SMX (10 ppm)	0.406	100% (60 min)	[4]
$CoFe_2O_4$-EG (0.6)	SMX (10 ppm)	0.4	100% (20 min)	[5]
$FeCo_2S_4$-CN (0.02)	SMX (5 ppm)	0.15	100 (15 min)	[6]
Fe_3O_4/β-FeOOH (0.2)	SMX (5 ppm)	0.24	100 (30 min)	[7]
Fe–Co–O–g-C_3N_4 (0.2)	SMX (10 ppm)	0.8	100 (30 min)	[8]
CuO@FeO_x@Fe (0.5)	SMX (10 ppm)	0.2	100 (10 min)	[9]
Fe@N-dopedcarbon (0.05)	SMX (15 ppm)	0.65	100 (20 min)	[10]
SA-Cu/rGO (0.1)	SMX (10 ppm)	0.4	100 (60 min)	[11]
Fe_3C@NCNTs (0.1)	SMX (5 ppm)	1	100 (60 min)	[12]
Fe-MOFs-MWs (0.5)	SMX (20 ppm)	9.6	100 (60 min)	[13]
CuO@Al_2O_3 (EPC) (0.5)	SMX (10 ppm)	0.4	100 (120 min)	[14]
Co/Co_9S_8@N-S-O-C (0.1)	SMX (10 ppm)	0.8	100% (10 min)	[15]

Table S9. Possible intermediate of SMX degradation.

Product ID and chemical formula	Proposed Structure	Measured accurate m/z
P1 ($C_{10}H_{11}N_3O_3S$)	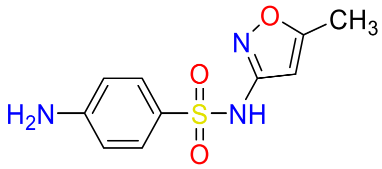	254.0603
P2 ($C_6H_8N_2O_2S$)	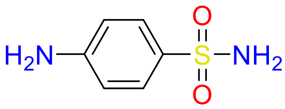	173.0405
P3 ($C_3H_6N_2O_4S$)	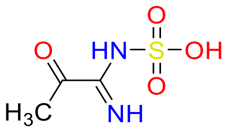	167.0125
P4 ($C_6H_5NO_2$)	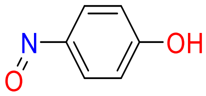	124.0410
P5 ($C_6H_6O_2$)	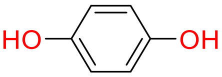	111.0398
P6 ($C_4H_6N_2O$)	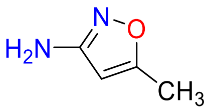	99.0556
P7 ($C_4H_7NO_2$)	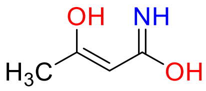	102.0340
P8 (C_6H_6O)	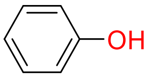	94.0453
P9 ($C_4H_6N_2$)	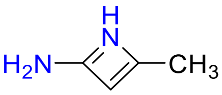	83.0607
P10 (C_2H_5NO)	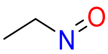	60.0451

Table S10. Theoretical computations of PMS adsorption onto different models.

Configuration	l_{O-O} (Å)	Q(e)	Eads (eV)
Free PMS	1.331		
Graphite-PMS	1.443	0.617	1.117
Graphitic N-PMS	1.475	0.959	2.121
Pyridinic N-PMS	1.439	0.576	1.036
Pyrrolic N-PMS	1.438	0.582	0.994
Fe (100)-PMS	1.484	0.997	2.687
FeO (100)-PMS	1.466	0.795	2.856
Fe_2O_3 (110)-PMS	1.467	0.674	3.263
Fe-N_4-Graphite-PMS	1.466	0.848	2.403

Table S11. Theoretical computations of $-NH_2$, -NH and $-CH_3$ adsorption onto different models.

Configuration	Total energy (eV)	Adsorption energy (eV)	Hydrogen bond length (Å)
Free SMX ($-NH_2$)	178.662		
Graphitic N-(SMX-NH_2)	699.152	-0.226	2.467
Pyridinic N-(SMX-NH_2)	699.278	-0.198	2.106
Pyrrolic N-(SMX-NH_2)	699.139	-0.352	2.624
Fe-N_4-(SMX-NH_2)	699.124	-0.374	1.999
Oxidic N-(SMX-NH_2)	699.301	-0.212	2.209
Graphitic N-(SMX-NH)	699.395	-0.468	2.764
Pyridinic N-(SMX-NH)	699.297	-0.370	2.793
Pyrrolic N-(SMX-NH)	699.461	-0.533	2.473
Fe-N_4-(SMX-NH)	699.313	-0.628	2.374
Oxidic N-(SMX-NH)	699.555	-0.387	2.660
Graphitic N-(SMX-CH_3)	699.162	-0.235	2.601
Pyridinic N-(SMX-CH_3)	699.085	-0.159	2.393
Pyrrolic N-(SMX-CH_3)	699.285	-0.338	2.436
Fe-N_4-(SMX-CH_3)	699.084	-0.359	2.357
Oxidic N-(SMX-CH_3)	699.265	-0.158	2.563

Reference

[1] R. Guo, L.-c. Nengzi, Y. Chen, Y. Li, X. Zhang, and X. Cheng, Chem. Eng. J. **2020,** 398.

[2] T. B. Nguyen, C. P. Huang, R.-A. Doong, C.-W. Chen, and C.-D. Dong, J. Hazard. Mater. **2021**, *401*, 123326.

[3] H. Zeng, L. Deng, H. Zhang, C. Zhou, and Z. Shi, J. Hazard. Mater. **2020**, 400.

[4] J. Zhang, W. Zhao, Z. Li, G. Lu, and M. Zhu, Chem. Eng. J. **2021**, 403.

[5] M. J. Xu, J. Li, Y. Yan, X. G. Zhao, J. F. Yan, Y. H. Zhang, B. Lai, X. Chen, and L. P. Song, Chem. Eng. J. **2019**, *369*, 403.

[6] Y. J. Li, J. Li, Y. T. Pan, Z. K. Xiong, G. Yao, R. Z. Xie, and B. Lai, Chem. Eng. J. **2020**, 384, 14.

[7] C. X. Li, J. E. Wu, W. Peng, Z. D. Fang, and J. Liu, Chem. Eng. J. **2019**, *356*, 904.

[8] S. Wang, Y. Liu, and J. Wang, Environ. Sci. Technol. **2020**, 54, 10361.

[9] Y. L. He, J. L. Zhang, H. Y. Zhou, G. Yao, and B. Lai, Chem. Eng. J. **2020**, *380*, 12.

[10] C. Liu, L. Y. Liu, X. Tian, Y. P. Wang, R. Y. Li, Y. T. Zhang, Z. L. Song, B. B. Xu, W. Chu, F. Qi, and A. Ikhlaq, Appl. Catal. B **2019**, 255, 11.

[11] F. Chen, X. L. Wu, L. Yang, C. F. Chen, H. J. Lin, and J. R. Chen, Chem. Eng. J. **2020**, *394*, 9.

[12] Y. N. Shang, C. Chen, P. Zhang, Q. Y. Yue, Y. W. Li, B. Y. Gao, and X. Xu, Chem. Eng. J. **2019**, 375, 12.

[13] Y. J. Wan, J. Q. Wan, J. R. Zhao, Y. Wang, T. Luo, S. Yang, and Y. X. Liu, *Chemosphere* **2020**, 254, 13.

[14] J. F. Yan, J. Li, J. L. Peng, H. Zhang, Y. H. Zhang, and B. Lai, Chem. Eng. J. **2019**, 359, 1097.

[15] S. Z. Wang, H. Y. Liu, and J. L. Wang, J. Hazard. Mater. **2020**, 387, 10.
